# Navigating bioactivity space in anti-tubercular drug discovery through the deployment of advanced machine learning models and cheminformatics tools: a molecular modeling based retrospective study

**DOI:** 10.3389/fphar.2023.1265573

**Published:** 2023-08-29

**Authors:** Ratul Bhowmik, Ravi Kant, Ajay Manaithiya, Daman Saluja, Bharti Vyas, Ranajit Nath, Kamal A. Qureshi, Seppo Parkkila, Ashok Aspatwar

**Affiliations:** ^1^ Medicinal Chemistry and Molecular Modelling Lab, Department of Pharmaceutical Chemistry, School of Pharmaceutical Education and Research, Jamia Hamdard, New Delhi, India; ^2^ Medical Biotechnology Laboratory, Dr. B. R. Ambedkar Center for Biomedical Research, Delhi School of Public Health, IoE, University of Delhi, Delhi, India; ^3^ Department of Bioinformatics, School of Interdisciplinary Studies, Jamia Hamdard, New Delhi, India; ^4^ Department of Pharmaceutics, School of Pharmaceutical Sciences, Siksha ‘O’ Anusandhan University, Bhubaneswar, Odisha, India; ^5^ Department of Pharmaceutics, Unaizah College of Pharmacy, Qassim University, Unaizah, Al-Qassim, Saudi Arabia; ^6^ Faculty of Medicine and Health Technology, Tampere University, Tampere, Finland; ^7^ Fimlab Ltd., Tampere University Hospital, Tampere, Finland

**Keywords:** molecular docking, tuberculosis, drug resistance, QSAR, pharmacophore modeling

## Abstract

*Mycobacterium tuberculosis* is the bacterial strain that causes tuberculosis (TB). However, multidrug-resistant and extensively drug-resistant tuberculosis are significant obstacles to effective treatment. As a result, novel therapies against various strains of *M. tuberculosis* have been developed. Drug development is a lengthy procedure that includes identifying target protein and isolation, preclinical testing of the drug, and various phases of a clinical trial, *etc.*, can take decades for a molecule to reach the market. Computational approaches such as QSAR, molecular docking techniques, and pharmacophore modeling have aided drug development. In this review article, we have discussed the various techniques in tuberculosis drug discovery by briefly introducing them and their importance. Also, the different databases, methods, approaches, and software used in conducting QSAR, pharmacophore modeling, and molecular docking have been discussed. The other targets targeted by these techniques in tuberculosis drug discovery have also been discussed, with important molecules discovered using these computational approaches. This review article also presents the list of drugs in a clinical trial for tuberculosis found drugs. Finally, we concluded with the challenges and future perspectives of these techniques in drug discovery.

## Introduction

Tuberculosis (TB) is a bacterial disease caused due to the infection of *Mycobacterium tuberculosis* (Mtb), which has been a chronic infectious disease for decades. According to the WHO, approximately 30 million persons are expected to be infected with the *bacillus* within 20 years (Vastrad, 2012)**.** It typically affects the lungs and other regions of the body, such as the spine, kidneys, and brain, if not treated swiftly. Tuberculosis (TB) has been proclaimed a global public health emergency by the WHO (Adeniji et al., 2018). As a result, discovering novel medications effective against MDR (Multidrug-resistant) TB, extensively drug-resistant TB (XDR TB), and latent TB is a key priority (Dwivedi et al., 2011; Gautam et al., 2023). Directly Observed Treatment Short is one of the most common anti-TB strategies (DOTS). This method, however, may be ineffective if performed incorrectly, leading to resistance to anti-TB drugs. MDR-TB will develop if anti-TB drug regimens are provided in incorrect dosages or with low-quality drugs (MDR-TB).

Moreover, if administered to treat individuals who are HIV-positive or have compromised immune systems, it could lead to the emergence of widespread drug-resistant tuberculosis (XDR-TB) (Shetye et al., 2020)**.** Isonicotinic acid, Hydrazide, Rifampicin, Ethambutol, Streptomycin, and other drugs have been used extensively in treating tuberculosis (Vastrad, 2012)**.** Although tuberculosis death is often preventable, the rapid rise in MDR and XDR-TB has necessitated the development of new drug targets for Mtb (Chapman et al., 2012)**.** TB is transferred mainly through the air when a healthy individual inhales these bacteria, which are droplets from air contaminated and take entry into the lungs. Either the host gets a primary infection, or the illness remains dormant. Alveolar macrophages perceive them as external agents who attempt to engulf the bacterium during this process. On the other hand, complete bacterium deactivation is nearly impossible to achieve. As a result, the bacteria multiply and infect macrophages, spreading to other parts of the lung **(** Ahamad et al., 2017; Jin et al., 2017)**.** This study aims to enhance anti-tubercular drug discovery by integrating advanced machine learning and cheminformatics tools. Using a molecular modeling-based approach, we aim to quickly identify potential drug candidates and targets against *Mycobacterium tuberculosis*, addressing drug-resistant strains. We’ll explore computational techniques like QSAR, molecular docking, and pharmacophore modeling to streamline drug discovery. Our goal is to predict, characterize, and prioritize drug molecules, including lead structures and novel targets, while assessing their versatility and utility. We’ll also highlight ongoing clinical trials and evaluate challenges and future prospects in computational drug discovery. Through these efforts, we aim to advance targeted therapies against drug-resistant tuberculosis using advanced computational methods**.**

### Mechanisms of action and limitations of antitubercular drugs

These drugs are categorized according to their source, like synthetic, semisynthetic, and natural products. Patient situation/stage (lines) and mode of action must be considered during treatment (Ahamad et al., 2017)**.** The finding and creation of new anti-TB therapeutics are widely recognized as one of the world’s most challenging public health issues; however, it is also a significant pharmaceutical challenge. Drug development is a lengthy procedure. Following a clinical trial, it can take decades for a molecule to reach the market. Computational approaches have aided drug development (Doreswamy and Vastrad, 2013; Ojo et al., 2021). The Quantitative Structure-Activity Relationship (QSAR) method is a powerful tool that is used in drug development all over the world. QSAR models are mathematical equations that show how chemical structures and biological processes are linked. The QSAR approach can potentially minimize the time and effort necessary to find novel compounds or increase the efficiency of current ones **(** Adeniji et al., 2018)**.** QSAR models are increasingly used with virtual screening and combinatorial libraries to predict the fate of physiologically active compounds (Ahamad et al., 2017)**.** It is possible to use this method in the development of future drugs**.** Increasing the speed of QSAR-related studies would facilitate the design and optimization of new drug candidates (Adeniji et al., 2018)**.** Another capability of these models is that they can provide a deeper understanding of biological activity mechanisms (Vastrad, 2012)**.** In QSAR modeling, various descriptors were employed, such as constitutional, geometrical, topological, quantum chemical, and other descriptors **(** Ojo et al., 2021)**.** This approach might be applied to predict the activity of newly proposed compounds before their synthesis and evaluation **(** Vastrad, 2012; Adeniji et al., 2018)**.** Molecular docking is a module that allows two or more molecules to recognize one other by matching their geometry and energy. It is a valuable tool in drug development for establishing the compatibility of molecules (ligands) with their target (receptor). It helps determine how a receptor interacts with its ligand and elucidates its binding process **(** Qing et al., 2014; Adeniji et al., 2018).

A pharmacophore must possess several chemical qualities to elicit a response from a receptor target. Pharmacophore models may be created using either a receptor alone or a receptor-ligand combination (Wermuth et al., 1998; Leach et al., 2010; Macalino et al., 2020). Pharmacophores are schematic representations of the main aspects of molecular recognition that may be used to represent and identify compounds on a 2D or 3D level. Physicophore model-based database screening is essential for computer-aided drug development since it gives information on receptor interaction’s geometric and electrical aspects (Qing et al., 2014). According to IUPAC, a Pharmacophore is a combination of steric and electronic qualities required for interaction with a target structure to trigger a biological response. Using a mix of pharmacophore model-based screening and docking studies to find novel drugs has been proven effective (Leach et al., 2010). Pharmacophore screening and docking can be combined to speed up the discovery of new drugs and improve their chances of survival (Macalino et al., 2020). Using molecular docking and other bioinformatic methods to evaluate candidate compounds before *in vitro* cell culture assays or chemical changes can help speed up drug discovery. Pharmaceutical and medicinal chemists can use QSAR and molecular docking investigations to design and synthesize novel anti-TB drugs (Adeniji et al., 2018)**.**

### QSAR, pharmacophore modeling, and molecular docking and their importance in drug discovery

The process of drug discovery and the development of a novel medicine is costly and time-consuming. Several laboratories and *in vivo* tests are used to determine therapeutic effectiveness and health risks. As a result, new methods are being developed to limit animal use in research, reducing ethical (and budgetary) concerns **(** Bajot, 2010).

The computational tools are mainly utilized to.

(i) perform the molecular structure confirmation (e.g., molecular dynamics);
(ii) characterize the interactions between drugs and targets (e.g., molecular docking);
(iii) to assess and optimize the activity of the drug through QSAR techniques.

**FIGURE 1:**
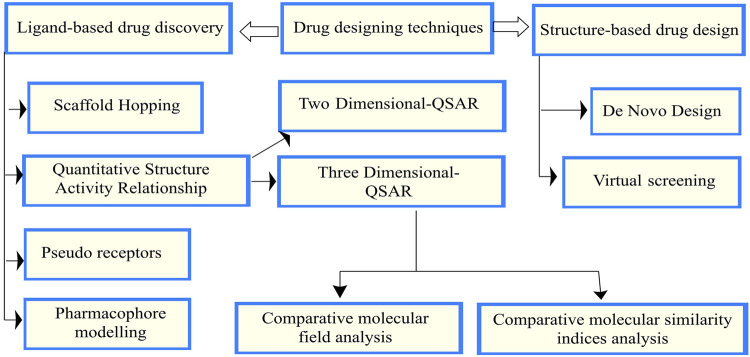
Drug design techniques and groups.

**FIGURE 2:**
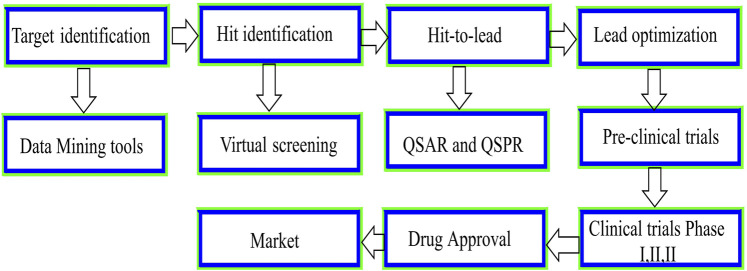
Schematic presentation of the drug discovery and development process.

There are mainly two drug design techniques: structure and ligand-based drug design (SBDD and LBDD). Using SBDD, any target inhibitor molecule can be designed, while LBDD primarily focuses on the chemical interaction between the target receptor and the inhibitor **(** Abdel-Ilah et al., 2017)**.** The drug design techniques and groups are presented in Figures 1, 2 (Abdel-Ilah et al., 2017).

## The significance of QSAR

**FIGURE 3:**
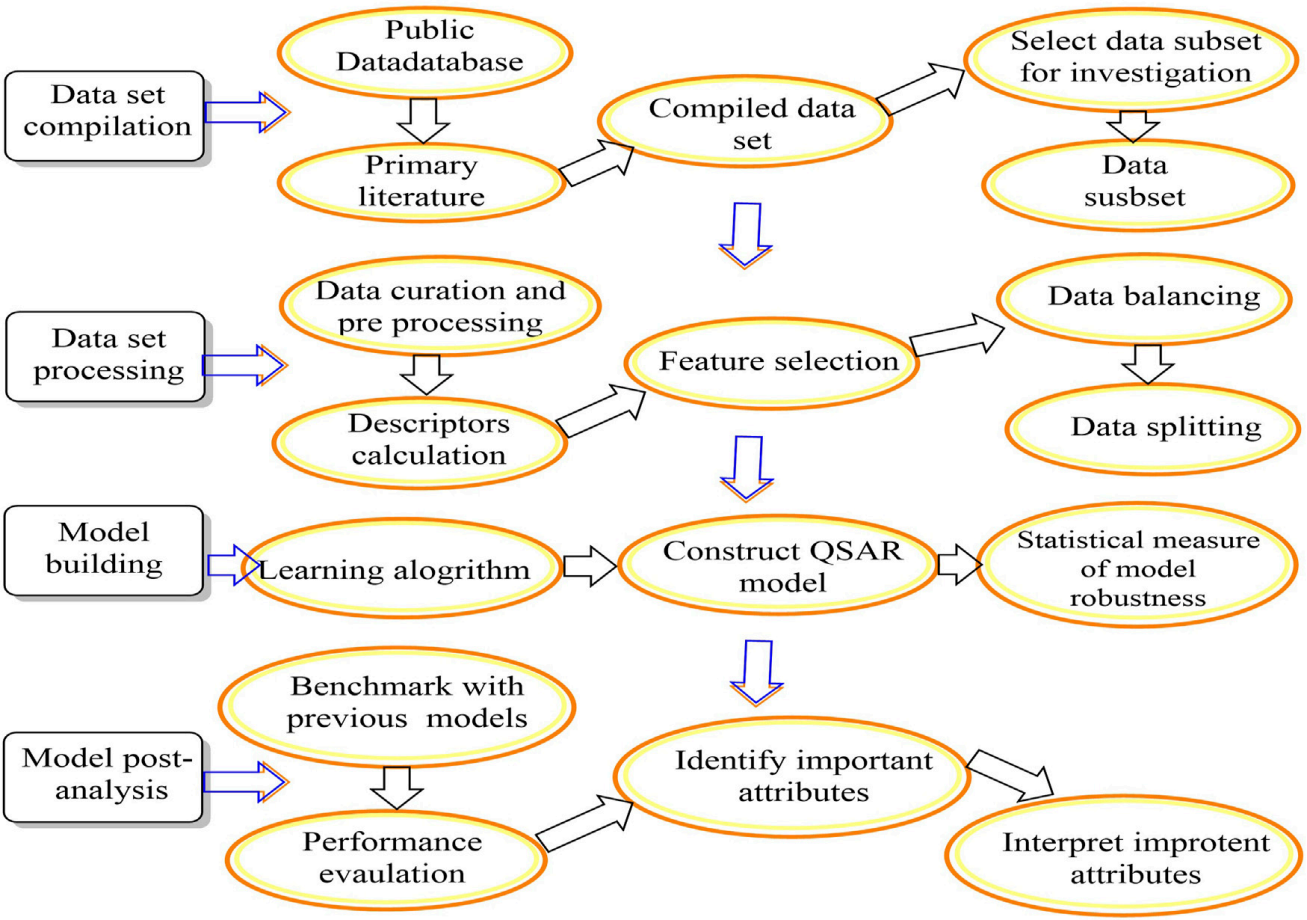
A workflow for QSAR modelling.

Quantitative structure-activity relationships (QSARs) utilize computational and mathematical models to identify the correlation between pharmacological activities and chemical structure compounds **(** Kwon et al., 2019)**.** QSAR is gaining traction as a less expensive alternative to medium-throughput *in vitro* and low-throughput *in vivo* research in the drug development process (Figure 2) (Prachayasittikul et al., 2015; Qureshi et al., 2023). In addition, QSAR models are increasingly used in drug discovery and environmental toxicology to predict and classify drug resistance, toxicity, and physicochemical characteristics (Verma et al., 2010). The QSAR technique is based on the premise that changes in a chemical’s molecular structure may be quantitatively linked to variations in its biological activity **(** Testa, 1995). Hammett first discovered the QSAR in the 1930s, and Hansch and Fujita developed it in the mid-1960s **(** Tandon et al., 2019)**.** Hammett’s works are significant contributions to the field of chemistry, particularly in the realm of Quantitative Structure-Property Relationships (QSPR). Through his research, Hammett established intricate mathematical connections between the acidity of compounds and the electronic effects of various functional groups. This innovative approach shed light on the underlying principles governing chemical reactivity and laid the groundwork for developing Quantitative Structure-Activity Relationship (QSAR) models. Medicinal chemists may now think about their structures in terms of physical properties rather than only pharmacophore groups due to the QSAR approach and philosophy. As a result of research, new inhibitors may be created from scratch, and existing medicines may be improved regarding absorption, distribution, metabolism, excretion, and toxicity (Abdel-Ilah et al., 2017; Kwon et al., 2019). QSARs are a computerized statistical method for explaining observed variation in replacement structure changes. QSAR modeling has extensively prioritized compounds for manufacture and biological assessment. The QSAR models may be utilized to identify potential hits and enhance hit-to-lead ratios, to aid in the efficient selection and optimization of compounds for further development and biological evaluation **(** Neves et al., 2018). Because no chemical needs to be made or tested before computer assessment, QSAR is a labor-, time-, and cost-effective technique for acquiring molecules with desired biological characteristics. As a result, QSAR is extensively employed in businesses, colleges, and research institutions throughout the globe **(** Cherkasov et al., 2014). There are five main steps in QSAR, including incorporating molecular structures and creating three-dimensional models. Since geometric descriptor calculations require molecular models in three dimensions: i) developing molecular structure descriptors, ii) selecting the most critical descriptors, which can be accomplished by using feature selection methods, iii) developing QSPR/QSAR models using the descriptor sets, and iv) validating the model by predicting the activity of substances based on external prediction data (Winkler, 2002) (Figure 3) **(** Piir et al., 2018).

Virtual screening (VS.) is a common computer approach for screening huge libraries of smaller molecules for novel hits with desirable features that may then be evaluated experimentally. Like other computational techniques, VS. aims to speed up the discovery process by minimizing the number of candidates that must be tested and rationalizing their selection (Neves et al., 2018). Furthermore, due to its time, cost, resource, and labor reductions, VS. has become quite popular in pharmaceutical businesses and academic institutions **(** Schaduangrat et al., 2020)**.** QSAR analysis is the most effective VS. technique due to its high throughput and hit rate **(** Aparoy et al., 2012). The Organization for Economic Cooperation and Development (OECD) accepted the following five principles for effective QSAR models to be used in regulatory evaluations of chemical safety: 1) a stated end aim; 2) a clear technique; 3) a defined scope of application; 4) appropriate goodness-of-fit, robustness, and predictability metrics; and 5), if feasible, a mechanistic interpretation **(** Gandhi et al., 2021)**.**

The Quantitative protein (or proteome)- disease relationships (QPDRs) are extensively utilized for illness prediction, whereas QSAR is frequently employed for pharmacological property prediction **(** Munteanu et al., 2010). Although they have been used for decades to predict and correlate the activity of molecules, there are several limitations to them, including i) the lack of training molecules in some cases; ii) they consider only two-dimensional structures; iii) the Hammett constant and other parameters are insufficient to link drug-receptor interactions; vi) there are no certain physiochemical criteria, no stereochemistry representation, and no one-of-a-kind solutions (Patel et al., 2014; Gandhi et al., 2021).

### Classification of QSAR methodologies

#### Based on the dimensionality of molecular descriptor

i. 0D QSAR- These descriptors are obtained from the molecular formula. 0D-QSAR focuses on zero-dimensional descriptors, such as constitutional descriptors, that include basic molecular information like the number of atoms, bonds, or functional groups (Gackowski et al., 2023).
ii. 1D QSAR- This correlates activity with global molecular parameters such as pKa, log P, and others. These descriptors are more straightforward and can be calculated more easily than higher-dimensional QSAR methods. The 1D-QSAR models are usually based on linear regression techniques, and they represent a straightforward approach to correlating molecular properties with biological activity 1D-QSAR has been applied to the study of various biological systems, such as the modeling of anti-cancer activity of a series of benzimidazole derivatives. This study’s models were based on simple one-dimensional descriptors, such as logP (partition coefficient), which provided significant insights into the molecular features responsible for the observed activity (Chandrasekaran et al., 2018).
iii. 2D QSAR- A molecular network containing topological or two-dimensional (2D) information is known as a 2D QSAR. The 2D-QSAR methodology involves the relationship between the chemical structure and biological activity of molecules, considering only two-dimensional properties like molecular weight, dipole moment, and hydrogen bond donors/acceptors. A classic application of 2D-QSAR is in drug discovery, where it has been used to model the activity of HIV protease inhibitors (Abdel-Ilah et al., 2017).
iv. 3D QSAR- These are calculated from a molecule’s geometrical or 3D representation. 3D-QSAR adds a third dimension to the analysis, considering the three-dimensional spatial arrangement of atoms in a molecule. It is often applied to understand how small molecules interact with a target protein in 3D space. 3D-QSAR has been extensively applied in studying enzyme inhibitors, such as developing new kinase inhibitors for cancer therapy (Ajjarapu et al., 2021).
v. 4D QSAR- This model describes four dimensions of information, with the fourth dimension being an ensemble of conformation for each ligand. 4D-QSAR includes the three spatial dimensions and adds the fourth dimension, representing molecular flexibility or time-dependent behavior. This considers how a molecule’s shape might change over time or under different conditions (Hopfinger et al., 1997).
vi. 5D QSAR- In 4D-QSAR, 5D-QSAR explicitly represents different induced-fit models (Verma et al., 2010; Patel et al., 2014; Abdel-Ilah et al., 2017). 5D-QSAR adds a fifth dimension, often representing the molecular solvation effects. It considers how solvent molecules interact with the molecule of interest (Good, 2006).

#### Based on the type of chemometric methods used

i. Linear method: Linear methods assume a linear relationship between the structure and activity. Common linear methods include multiple linear regression (MLR), partial least squares (PLS), and principal component regression (PCR).
ii. Non-linear method: Non-linear methods are used when the relationship between the structure and activity is complex and non-linear. Techniques include artificial neural networks (ANNs), support vector machines (SVMs), and k-nearest neighbors (k-NN).

### Types of techniques for QSAR modeling

1. **The simple linear regression (SLR)** method generates a QSAR model in equations using a standard linear regression calculation. This technique has proven to be quite promising for developing structure and activity correlations (Verma et al., 2010). Using a straight line, SLR models the relationship between a single independent variable and a dependent variable. It assumes a linear relationship and is widely used for prediction and understanding how the variables are related. The method is simple, interpretable, and widely applied in various scientific fields. SLR might not capture complex relationships that involve multiple variables (Draper and Smith, 1998). Linear Methods, Used in modeling structure-activity relationships in drug discovery, predicts novel compounds’ biological activity (Krishnapuram et al., 2005)**.**
2. **Multiple linear regression (MLR)** extends SLR to several dimensions. Standard multivariable regression calculations are used in this procedure. All of the descriptors under study are subjected to identifying a drug property. MLR provides a more nuanced understanding of the system under study by considering more variables. It is a powerful tool for prediction and explanation but requires careful handling of collinearity among predictors. Adequate variable selection is essential for building meaningful models (Schneider et al., 2010 **).**
3. **Stepwise multiple linear regression-** Variation MLR, which yields a multiple-term linear equation but does not use all independent variables, is widely used in this approach. This method works effectively when there are a lot of descriptors and the key ones are not known (Patel et al., 2014)**.** SMLR combines the principles of MLR with a sparsity constraint, ensuring that only the most relevant variables are used in the model. This can lead to better interpretability and prevent overfitting. SMLR is particularly useful when dealing with high-dimensional data requiring feature selection. It is a modern technique that bridges statistical modeling with machine learning (Krishnapuram et al., 2005).
4. **The partial least square method (PLS)** provides a statistically robust solution even when the independent variables are heavily connected, or the number of observations exceeds that **(** Verma et al., 2010; Patel et al., 2014)**.** PLS is a sophisticated regression method that extracts latent variables explaining the covariance between independent and dependent variables. It is widely used in chemometrics for handling multicollinearity. PLS provides robust and interpretable models by focusing on the variables most related to the response. It is precious when numerous predictors are highly correlated (Mehmood et al., 2020). Applied in various fields like drug design, metabolomics, and environmental toxicity prediction, where non-linear relationships are common (Everitt et al., 2011).
5. **Principle components analysis (PCA)** is a technique for creating a new set of orthogonal descriptors called principal components (PCs) that describe the bulk of the information in the independent variables in decreasing order of variance. CA is also utilized in PLS approaches for variable selection (Patel et al., 2014)**. P**CA is a dimensionality reduction technique that transforms the data into orthogonal components, capturing the most variance. It is a robust exploratory data analysis, visualization, and preprocessing tool. PCA helps understand the underlying structure of data and is widely used in various scientific fields, including chemometrics and bioinformatics (Prachayasittikul et al., 2015; Jolliffe and Cadima, 2016).
6. **The genetic function approximation (GFA)** method can be used as an alternative to average regression analysis to construct QSAR equations. It can create both linear and higher-order non-linear equations. Genetic algorithm partial least squares (G/PLS or GA-PLS) are a helpful method that combines the most significant features of GFA and PLS. GFA applies genetic algorithms to find the optimal subset of descriptors in regression analysis. It can model complex non-linear relationships and is highly flexible. GFA has been applied successfully to model various chemical and biological systems, especially in QSAR studies. Its ability to navigate vast descriptor spaces makes it a valuable tool in computational chemistry (Rogers and Hopfinger, 1994).
7. **Cluster analysis** is a multivariate approach for classifying structures into subsets (called clusters) that are similar in some manner (Patel et al., 2014)**.** Cluster analysis is a grouping technique used to categorize objects into clusters based on their similarity. It is unsupervised, meaning that the categories are not predefined. Cluster analysis has diverse applications, including market segmentation, image processing, and pattern recognition. It provides insights into the natural groupings within data (Zhang et al., 2023).
8. **Artificial neural networks (ANNs)** are nonlinear computational models that simulate the activity of human neurons to make predictions **(** Prachayasittikul et al., 2015). They can be used to model QSAR and solve pattern recognition difficulties (Patel et al., 2014). ANNs are inspired by the human brain’s function and consist of interconnected nodes or neurons. They are capable of modeling complex, non-linear relationships between inputs and outputs. ANNs have been applied in numerous fields, including image recognition, natural language processing, and QSAR modeling. The flexibility and adaptability of ANNs make them a powerful tool, but they require careful tuning and interpretation (Trinh et al., 2021). Used in virtual screening and toxicity prediction, providing accurate models that can handle the complexity of biological systems (Pérez et al., 2021).

### Molecular descriptors and their significance

Despite significant advancements in drug design, descriptors used to designate the molecular structure of biologically active compounds remain the primary method for identifying novel lead molecules. For QSAR/QSPR investigations, descriptors are numerical representations of the chemical properties of a molecule. For statistical model construction to be possible, the mathematical representation of these descriptors must be independent of the molecule’s size and number of elements. In QSAR/QSPR modeling, molecular descriptors have evolved into the most important variables. The information conveyed by descriptors typically depends on the type of molecular representation and the defined algorithm for its calculation. Among these are topological indices, geometrical, structural, and physicochemical descriptors.

Constitutional descriptors are basic, widely-applied descriptors that reflect the molecular composition of a compound without providing information about its topology. The most common constitution descriptors are the number of atoms, number of bonds, variety of atoms, ring count, and molecular weight (MW). These descriptors are insensitive to conformational changes and do not distinguish between isomers.

Recent developments in lead discovery, drug design, virtual screening, combinatorial library design, and database search discrimination also highlight the importance of topological descriptors in drug discovery. Topological indices (TIs) are two-dimensional descriptors that consider the intrinsic atomic arrangement of compounds. These descriptors are derived from the topological representation of molecules and can be considered structure-specific. These indices encode numerical information regarding the molecular size, shape, branching, presence of heteroatoms, and number of bonds. By the nature of chemical bonds, these TIs represent the interconnectedness of atoms within molecules. They play a crucial role in modeling various physicochemical properties, biological activities, and pharmacokinetic properties. A molecular graph represents a topological representation of a molecule. This graph is denoted mathematically as G = (V, E), where V is the set of vertices corresponding to the atoms of the molecule, and E is the set of elements representing the binary relationship between pairs of vertices. These chemical graphs depict the molecular structure in a non-numerical format; however, a numerical translation of the graph is required to calculate topological descriptors. The most commonly used descriptors are the Wiener index, the Connectivity indices, the Kier shape, the Balaban J index, and the Zagreb indices. The primary function of these indices is to classify molecules according to their size, degree of branching, flexibility, and overall morphology (Wiener, 1947; Randic, 1975; Balaban, 1982; Roy, 2004).

The 3D coordinates of the atoms in a given molecule are used to derive geometrical descriptors. In comparison to topological descriptors, these descriptors are abundant in information and discrimination power for analogous chemical structures and molecule conformations. In addition, they contain data acquired from atomic van der Waals regions and their overlap on the molecular surface. Despite their high informational density, these descriptors typically have disadvantages as well. Geometric descriptors necessitate geometry optimization and, consequently, the computational burden to calculate them. Thus, for flexible molecules that can assume multiple conformations, new information can be utilized. Nonetheless, this results in the issue that complexity can increase substantially. Moreover, alignment constraints are required for the majority of these descriptors (grid-based descriptors) to accomplish molecule comparability. A molecule’s physical and chemical properties that can be estimated from its two-dimensional structure are physicochemical descriptors. These properties play a significant role in determining the substance concentration in the body. A drug’s efficacy and, consequently, its market value can be enhanced by its possessing the appropriate properties. Thus, examining these properties of a drug not only contributes to the drug’s safety profile but also plays a crucial role in drug discovery by optimizing the compounds chosen. In addition to selecting candidate compounds with the appropriate physicochemical properties, it is necessary to pay special attention to properties such as lipophilicity, solubility, and permeability, which can ensure optimal potency (Leo et al., 1975; Ghose and Crippen, 1986). In contrast, molecular fingerprints have been utilized for decades to investigate large chemical libraries for similar compounds. The information content of 2D signatures is derived from atoms, bond types, and graph distances derived from chemical graphs, where these are represented as bits. Each bit indicates the presence or absence of a predetermined substructure in a compound. A bit in a structural fingerprint corresponds to a chemical property, typically the presence of some substructure. Based on the similarity to a biologically active molecule, these biomarkers enable researchers to identify additional compounds with a higher probability of displaying similar biological potency against the same target. Molecule properties, such as chemical diversity in chemical space, can be characterized using fingerprints. Such evaluations are crucial in the compound selection process before experimental screening. Numerous fingerprint varieties can be used for structural comparisons of various sorts. Such signatures have become a popular option for drug discovery because they offer a decent balance and empirically reasonable proportionality. By quantifying fingerprint overlap with similarity coefficients and using the resulting values to measure molecular similarity, fingerprint similarity searching generates a database ranking. A Tanimoto coefficient (Tc) value of >0.85 indicates a high probability that the test compounds have similar bioactivity. However, Tc is only useful for data with comparable levels of complexity; other coefficients are preferred for data with varying levels of complexity (Willett, 2006; Rogers and Hahn, 2010; Helal et al., 2016).

Initially, 2D fingerprints were designed for similarity searching using a single template, but some studies claim that search performance is enhanced when multiple reference compounds are employed. MACCS, PubChem, and Extended-Connectivity biometrics (ECFP) are the most commonly used biometrics. MACCS is a compilation of 166 bits that encompasses most of the chemical characteristics important for virtual screening. The PubChem signatures database contains 881 bits of descriptors for element counts, aromatic or nonaromatic ring counts, atom pairs, atom neighborhoods, and particular fragments. These 2D signatures have been successfully utilized in the virtual screening of novel active compounds. Using PubChem bioassays, comprehensive bioactivity profiles, dubbed “PubChem high-throughput screening fingerprints” (PubChem HTSFPs), were recently developed. In addition, these PubChem-HTSFPs were utilized in hit expansion experiments for 33 unique targets. These signatures were useful for retrieving matches with structural diversity and the desired bioactivities. ECFPs are a novel class of 2D circular signatures used for molecular characterization. These signatures are an extension of the Morgan algorithm. These circular fingerprints have numerous advantageous characteristics, including i) being easy to calculate; ii) representing a large number of distinct features; and iii) not being reliant on predefined features; thus, they can represent novel structural variation. These fingerprint types effectively employ encoded rich data for similarity searching, compound clustering, and chemical library analyses. In addition, ECFPs are frequently employed in QSAR and QSPR model development for lead optimization and ADMET property forecasting (Willett, 2006; Rogers and Hahn, 2010; Helal et al., 2016).

Therefore, concerning anti-tubercular drug discovery, a combination of different machine-learning-assisted QSAR models is required while implementing molecular descriptors and molecular fingerprints of molecules’ datasets for feature selection against any biological target. The best way to deal with major demerits of different types of molecular descriptors is to include two or more molecular descriptors while constructing the hybrid descriptors-based QSAR models. Hybrid molecular descriptors incorporate multiple molecular characteristics, including topological, electronic, and geometric properties. This exhaustive representation accounts for a broader range of factors that contribute to the activity of a molecule, thereby enhancing the model’s predictive accuracy. The same approach should be applied while considering molecular fingerprints for anti-tubercular drug design by constructing a hybrid fingerprint-based QSAR. Therefore, the use of both hybrid molecular descriptors and molecular fingerprint-based QSAR models will have a distinct and interpretable relationship with the activity of a molecule that will additionally aid researchers in analyzing the contributions of particular characteristics or substructures to the overall activity, thereby facilitating the design of novel compounds with the desired properties. Apart from this, hybrid molecular descriptors and molecular fingerprints can represent a wide variety of chemical structures, including novel and unconventional compounds. This adaptability is essential for investigating new chemical space and identifying potential drug candidates with distinctive structural characteristics. In ensemble modeling, where multiple QSAR models or screening methods are combined to improve predictive performance, hybrid molecular descriptors and molecular fingerprints can also be used. This strategy combines the advantages of various techniques, resulting in more accurate and robust forecasts. Due to their ability to provide a comprehensive, interpretable, and computationally efficient representation of molecular structure and activity, hybrid molecular descriptors and molecular fingerprints are favored for QSAR modeling and drug design. These techniques have significantly accelerated the drug discovery process by allowing researchers to prioritize and design promising compounds for further experimental evaluation (Willett, 2006; Rogers and Hahn, 2010; Helal et al., 2016). As computational methods continue to advance, hybrid descriptors and molecular signatures will likely play a greater role in shaping the future of drug development against tuberculosis.

### Modeling QSAR using chemical space analysis

Chemical space is vital in drug development for various purposes, including library creation, compound classification, selection, structure-activity relationship (SAR) investigation, and understanding structure-property connections (SPR) (Naveja Medina, 2019). It encompasses all descriptors derived from chemical compounds. The chemical space analysis for FDA-approved drugs showed shared traits, including substantial halogen content and molecular weights below 500, consistent with Lipinski’s rule of five. This rule suggests that drugs with an MW less than 500 have better bioabsorption and bioavailability (Prachayasittikul et al., 2015). Chemical space represents the collection of all potential organic compounds, and as its scale increases, cartographic approaches are used to visualize and conceptualize it. Machine learning methods benefit from chemical space approaches as they identify clusters of related compounds. Lipinski’s rule of five indicates that drug-like compounds exhibit drug resemblance with MW < 500 Da, clogP <5, few H-bond donors (<5), and few H-bond acceptors (<10) (Ganesan, 2008). Optimizing this rule is essential for improving BBB permeability by passive diffusion. Factors like MW, log P, and H-bond donors and acceptors influence drug interaction with the blood-brain barrier (BBB) (Lambrinidis and Tsantli, 2018). By analyzing the chemical composition of natural compounds and determining their molecular structures, the investigation identified 11 novel inhibitors for ß-hydroxysteroid dehydrogenase type 1 **(** Koch et al., 2005)**.** According to Reayi, diversity-oriented synthesis (DOS), a chemical synthesis strategy for swiftly building a library of compounds, can help deorphanize druggable protein targets **(** Reayi and Arya, 2005). ChemGPS-NP was used to examine the chemical space of natural products from different databases (Larsson et al., 2005). They discovered that 40,348 compounds from the Dictionary of Nature Products Database passed Lipinski’s rule of five **(** Rosén et al., 2009)**.** To define “bioactive natural compound-likeness,” (Zhou et al., 2010), used structure-activity relationships to investigate the chemical space of natural products, comparable to Lipinski’s rule of five (drug-likeness) (BNC-likeness). The drug-likeness and BNC-likeness models were utilized to compare the structural properties of bioactive and non-bioactive natural products. The Ethnobotanical Database and Dr. Duke’s Phytochemical Database were used to create a dataset of 1,580 natural products from a total of 7,549 natural product constituents. 790 natural compounds were bioactive, while the remaining 790 were not, resulting in a well-balanced dataset. Bioactive natural compound-likeness models were created using SVM with radial basis function kernels, and the training set consisted of 1,580 bioactive substances. An independent external data set of 81 bioactive and 81 non-bioactive natural compounds from commonly used medicinal plants were used to test the models’ performance. The prediction results effectively classified 75 bioactive chemicals, indicating the robustness of the models and their immunity to overfitting. One of the issues with machine learning is overfitting, which arises when noise data is used as an independent variable in developing highly predictive models **(** Zhou et al., 2010)**.**

### Model validation methodology for QSARs

**FIGURE 4:**
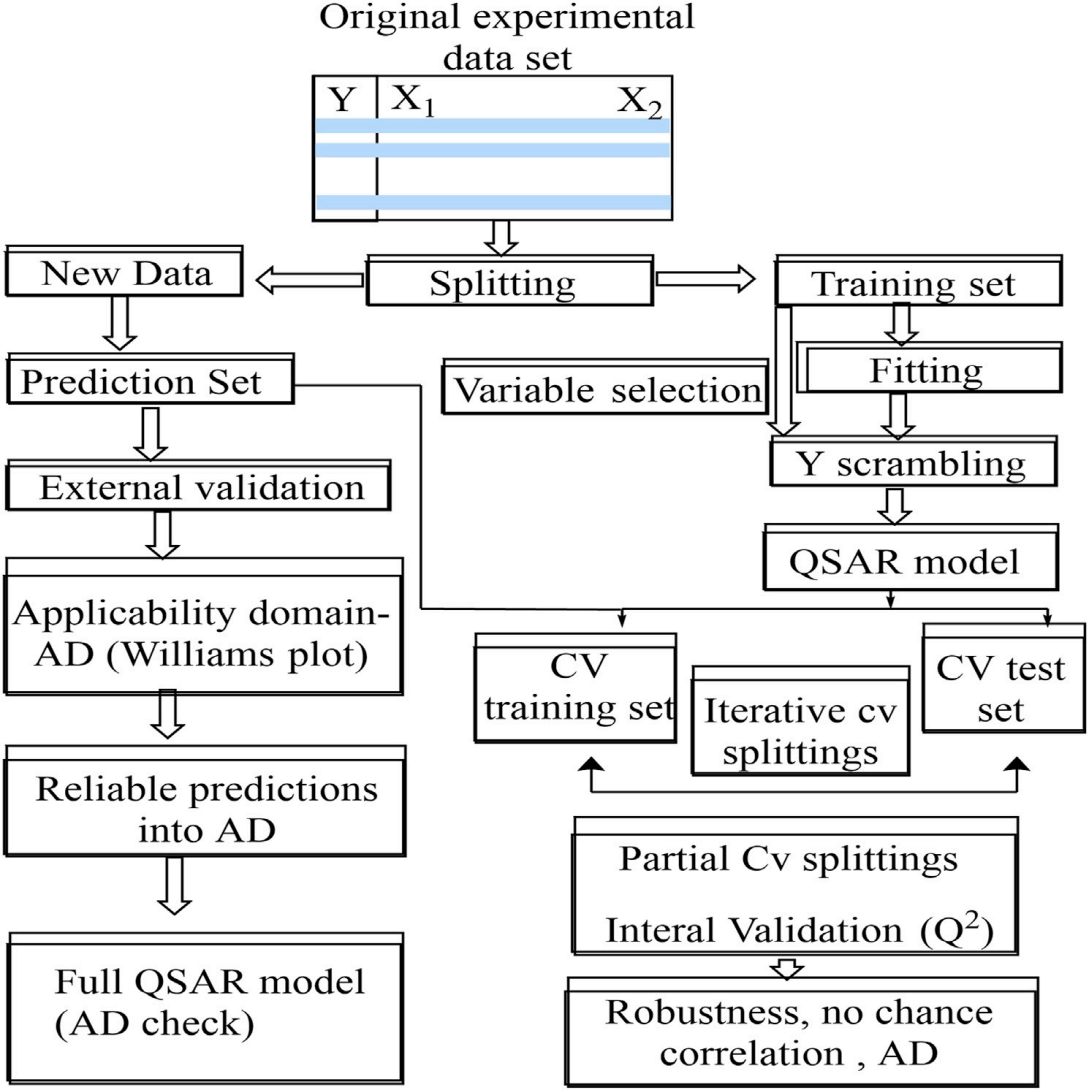
Predictive approach for QSAR modelling.

**FIGURE 5:**
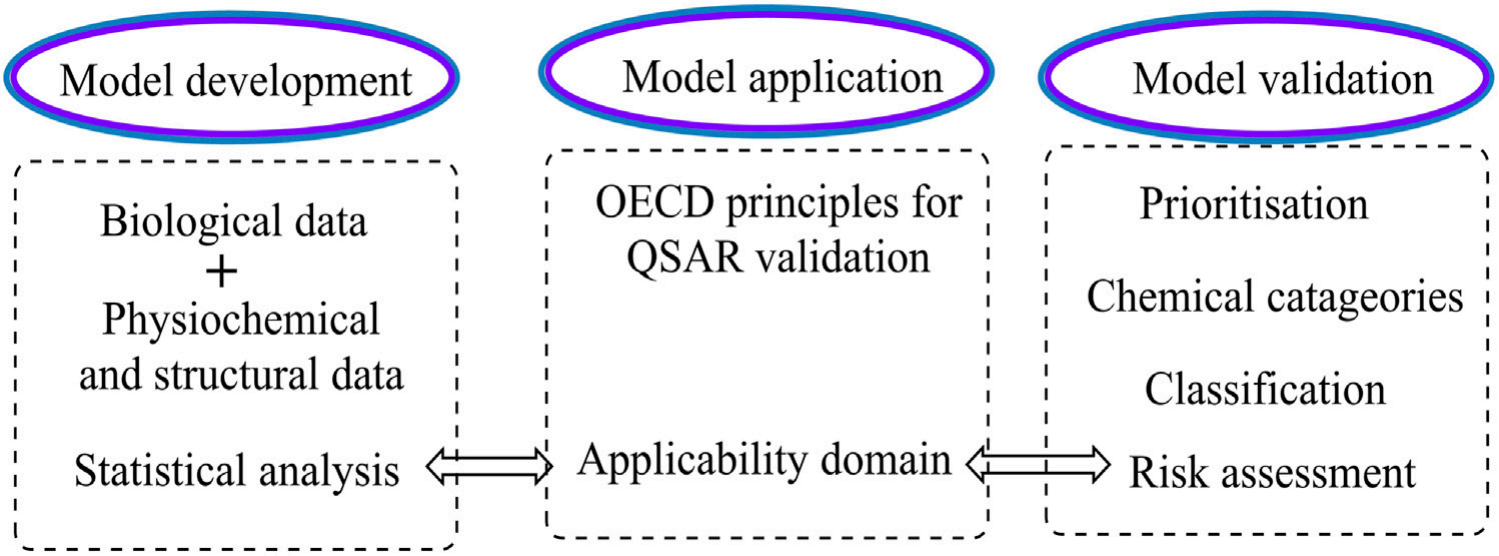
The role of applicability domain in various stages of the QSAR life cycle.

The validation approach tries to provide a model with defined descriptors that is statistically trustworthy because of a cause-and-effect connection rather than a random numerical link. Validation procedures are required to determine a model’s predictive ability. Internal and external validation techniques are the two sorts of validation methods accessible. Training datasets are used by internal techniques such as Q^2^ (squared correlation coefficient), *R* ^2^ (coefficient of determination or coefficient of multiple determination for multiple regression), chi-squared (X^2^), and root-mean-squared error (RMSE). When applied to new data sets, the model’s lack of predictability is a significant flaw in this technique. External approaches, on the other hand, are based on the testing set and are considered the most reliable validation **(** Abdel-Ilah et al., 2017; Kwon et al., 2019)**.** These statistical methods ensure that the models produced are accurate and unbiased. Cross-validation approaches such as accuracy (ACC), sensitivity (SEN), specificity (SPEC), and Matthew’s correlation coefficient (MCC) **(** Prachayasittikul et al., 2015) can be used to assess the model’s internal predictive capability. In contrast, its external predictability can be evaluated using a separate set of molecules (the test set) that were not used in the model creation **(** Testa, 1995; Verma et al., 2010). The CV method begins by removing one or more compounds from the training set, which serves as a temporary test set. The remaining data points are used to form a CV model, which is then tested on the deleted molecules to see if it can accurately predict bioactivities using the descriptors from the original model (Figures 4, 5).

External validation, the Y-randomization test, the domain of applicability, and the William plot are some statistical techniques that can be used to assess a QSAR/QSPR model’s predictive ability. External validation is a method of testing a QSAR/QSPR model’s external predictivity by omitting a section of data at the start of the experiment and using the remaining internal set to evaluate optimal learning algorithm parameters **(** Prachayasittikul et al., 2015)**.**

The accuracy (ACC), sensitivity (SEN), specificity (SPEC), and Matthew’s correlation coefficient MCC) are used to evaluate the prediction performance of the proposed QSAR/QSPR model using cross-validation (CV), which is given by the following formula Eqs 1–4

$$Accuracy=\frac{TP+TN}{\left(TP+TN+FP+FN\right)}\times 100$$ (1)

$$Sensitivity=\frac{TP}{\left(TP+FN\right)}\times 100$$ (2)

$$Specificity=\frac{TN}{\left(TN+FP\right)}\times 100$$ (3)

$$MCC=\frac{TP\times TN-FP\times FN}{\surd \left(TP+FN\right)\left(TN+FP\right)\left(TN+FN\right)}$$ (4)

TP, TN, FP, and FN- Numbers of true positive, true negative, false positive, and false negative.

Matthew’s correlation coefficient (MCC) measures the gap between actual and expected values. The coefficient is a balanced measure that can be employed when the classes are of various sizes and take into consideration true negatives (TN), true positives (TP), false negatives (FN), and false positives (FP). The equation is used to derive the formula. In machine learning, Matthew’s correlation coefficient (MCC) is used to assess the quality of binary and multiclass classifications. Like most correlation coefficients, MCC has a range of 1 to 1, with 1 denoting the best agreement between actuals and forecasts, −1 denoting an inverse prediction, and 0 denoting no agreement. Alternatively, the prediction is random in comparison to the actual situation **(** Mun and Geng, 2019)**.**

### Machine learning drug development

Due to an influx of available data and increased computer capacity, machine learning (ML) technologies are coming back in drug development investigations. This has sparked a flurry of artificial intelligence (AI) drug development investigations, in which machine learning (ML) and deep learning (DL) approaches are used to solve issues efficiently and intelligently. Combining structural, sequence, and evolutionary data yields machine-learning models (Verma et al., 2010)**.** An analysis of hydrophobicity, side-chain pKa, solubility, solvent accessibility, and other physiochemical parameters is conducted, as well as the development of a machine-learning model to predict binding and non-binding residues. Only a few models predict binding sites based on one of the binding partners’ structures, whereas others employ information from both partners **(** Kumar et al., 2018)**.** The two types of machine learning methodologies are unsupervised and supervised learning. Labels are allocated to training data in supervised learning, and the model can predict labels for specific data inputs once it is ready. On the other hand, unsupervised machine learning algorithms can profit directly from unlabeled molecular pattern data, as they require input data and no output components (Supplementary Table S7) (Singla et al., 2013).

### The significance of pharmacophore

**FIGURE 6:**
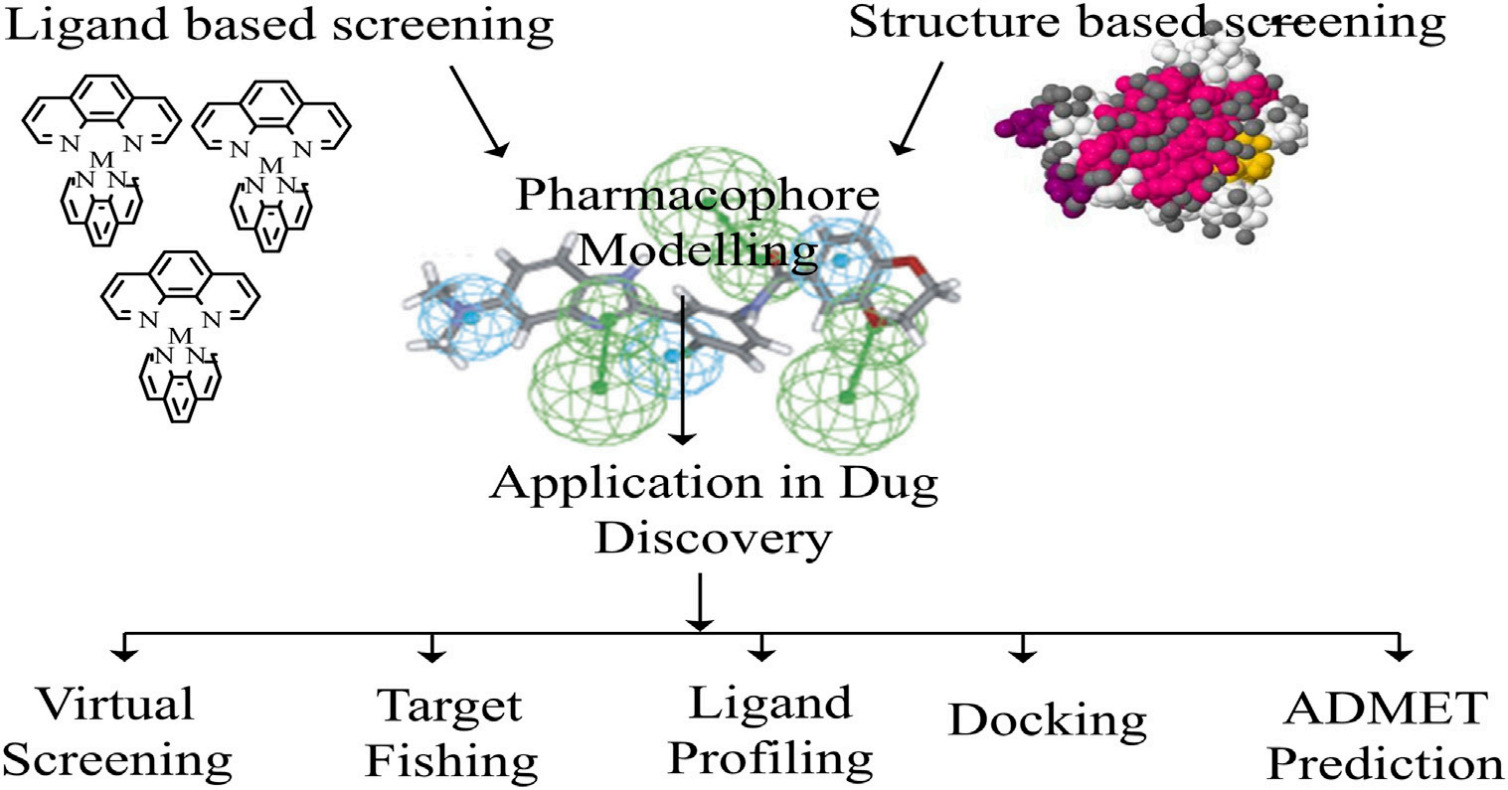
A framework of Pharmacophore architecture.

Ehrlich coined the pharmacophore as “a molecular framework that conveys (phoros) the key properties responsible for a drug’s (pharmacon) biological activity” in 1909 **(** Yang, 2010)**.** According to the IUPAC nomenclature (Leach et al., 2010), a pharmacophore is a molecule that possesses both steric and electronic properties that enable it to produce efficient supramolecular interactions with a biological target structure and activate or inhibit its biological response (Figure 6).

To better understand ligand-protein interactions, pharmacophore models are developed. They may be utilized to discover new compounds that match the pharmacophore criteria and are thus expected to be active **(** Muhammed and Aki-Yalcin, 2021)**.** Using a mix of pharmacophore model-based screening and docking studies to find new drugs has been proven beneficial **(** Mesli et al., 2021)**.** If the target’s structure is unknown, a pharmacophore model can be created utilizing structural data from the active ligands that bind to it. The ligand-based pharmacophore modeling technique (Yang, 2010; Muhammed and Aki-Yalcin, 2021) is the name given to this approach.

When the target’s structure is known, pharmacophore models can be constructed utilizing the target’s structural attributes. This is the structure-based pharmacophore modeling approach (Vastrad, 2012)**.** A whole framework of Pharmacophore is shown in Figure 6 (Muhammed and Aki-Yalcin, 2021). A list of pharmacophore modeling software is enlisted in (Supplementary Table S8) (Yang, 2010; Singla et al., 2013; Kumar et al., 2018; Schaller et al., 2020; Muhammed and Aki-Yalcin, 2021).

The pharmacophore analysis has the most important common denominator of the molecular interaction properties shared by a group of active compounds. It is an abstract idea rather than a physical molecule or combination of chemical groups **(** Qing et al., 2014)**.** Integrating knowledge about the three-dimensional nature of molecular interactions is another significant component of current pharmacophore research. This point of view is centered on 3d-pharmacophore techniques (Leach et al., 2010)**,** which define the spatial relationship between pharmacophore features. Virtual screening, *de novo* design, lead optimization, and multitarget drug design have all utilized pharmacophore-based approaches **(** Yang, 2010; Kumar et al., 2018; Muhammed and Aki-Yalcin, 2021)**.** There are limitations with pharmacophore scoring functions that limit its ability to realize its intended potential, especially with the current high cost of discovering and developing new medicine. The involvement of other computational methods is critical in overcoming these obstacles. As a result, combining pharmacophore modeling with different computational approaches addresses some of these constraints **(** Yang, 2010; Kumar et al., 2018)**.**

A pharmacophore model is comprised of several characteristics that are arranged in a three-dimensional (3D) pattern. The characteristics can be labeled as a single feature or any logic combination consisting of “AND,” “OR,” and “NOT” **(** Qing et al., 2014) to blend varied interaction patterns under a single label. Hydrogen bond donors (HBD), hydrogen bond acceptors (HBA), positive features, negative features, aromatic rings, hydrophobic features, and their combinations are all examples of molecular pharmacophore patterns **(** Seidel et al., 2020)**.** Different compounds can be compared at the pharmacophore level, known as “pharmacophore fingerprinting.” The pharmacophore is a “query” when only a few pharmacophore properties are analyzed in a 3D model **(** Qing et al., 2014)**.** Classification of 3D-QSAR is given in Supplementary Table S4. Pharmacophoric characteristics may be used as a query to search for possible leads from chemical compound databases and produce compounds with specific qualities (lead optimization) **(** Qing et al., 2014; Kumar et al., 2018; Muhammed and Aki-Yalcin, 2021)**.** It also uses pharmacophore fingerprints to determine the comparability and diversity of drugs. It may also align molecules based on their three-dimensional arrangement or create a predicted three-dimensional QSAR model **(** Kumar et al., 2018)**.** The webservers or databases for QSAR drug design research are given in Supplementary Table S1-3). A list of software used for calculating descriptors and fingerprints is presented in Supplementary Table S5, 6.

Pharmacophore modeling employs organized methods to construct a logical framework to identify other chemical moieties with similar properties against the disease’s target of interest. The steps are as i) ligand synthesis, ii) mapping of pharmacophore features, iii) looking for a common pharmacophore, and iv) calculating the shared pharmacophore score **(** Kumar et al., 2018). A pharmacophore can be used to screen new target-specific agonists and antagonists, toxicants, undiscovered targets, and the best molecular docking findings. A pharmacophore model is often used in virtual screens to locate medications that trigger the required biological activity (Yang, 2010; Muhammed and Aki-Yalcin, 2021)**,** as well as fishing drug targets, ligand profiling, docking, and ADMET (absorption, distribution, metabolism, excretion, and toxicity) prediction **(** Leach et al., 2010; Yang, 2010; Qing et al., 2014; Muhammed and Aki-Yalcin, 2021)**.**

### An overview of the importance of molecular docking

**FIGURE 7:**
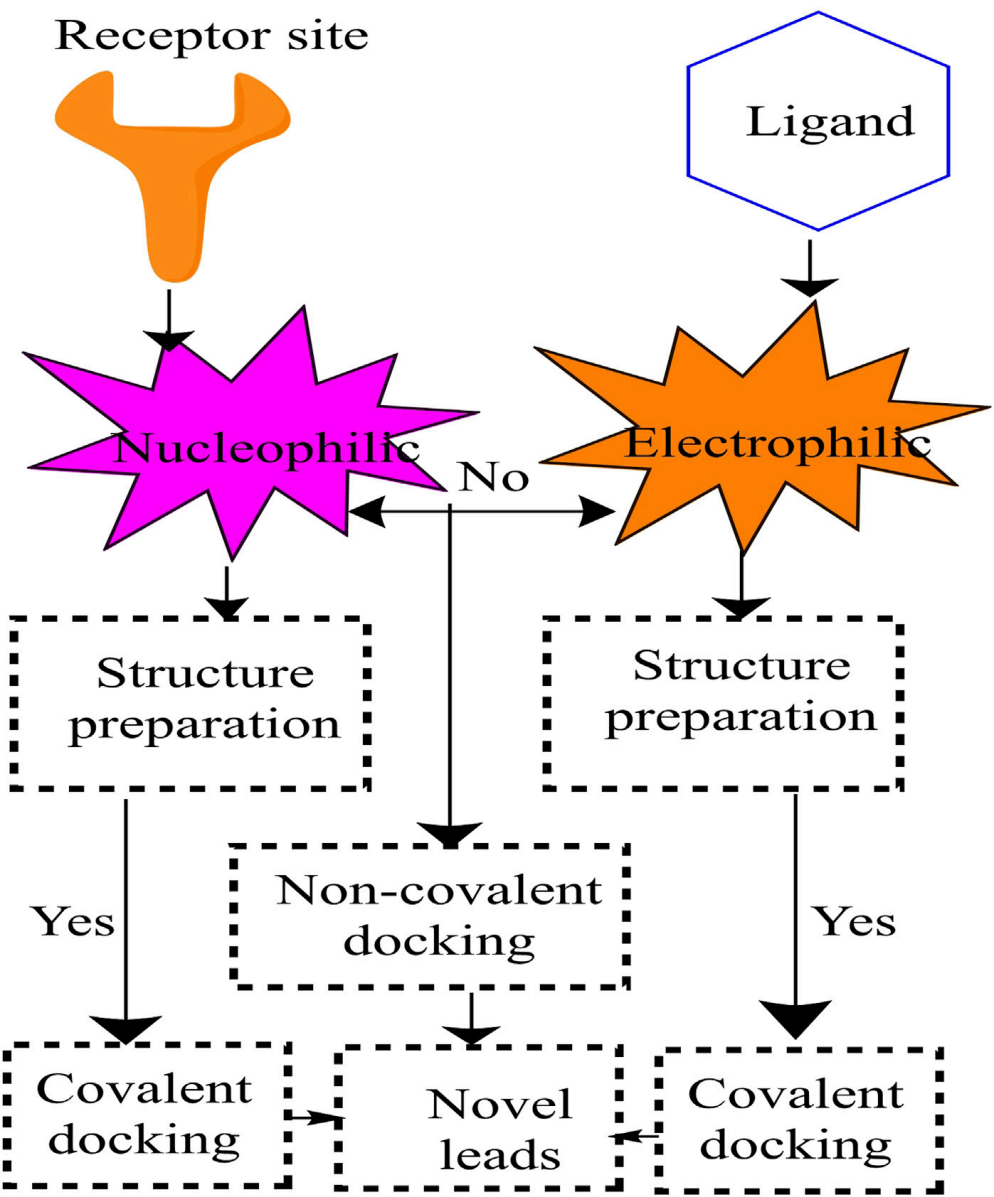
Schematic presentation of covalent docking.

The most extensively used strategy for structure-based drug development is molecular docking, which has been around since the early 1980s **(** Meng et al., 2012)**.** Molecular docking provides an attractive framework for studying drug biomolecular interactions, which is useful for rational drug design and discovery Dar and Mir, 2017)**.** The molecular docking approach may be used to model an atomic-level interaction between a small molecule and a protein, allowing us to characterize small molecule behavior at target protein binding sites and highlight key biochemical processes **(** Meng et al., 2012; Aspatwar et al., 2019) (Figure 7) (Ferreira et al., 2015).

When the target protein’s 3D structure is available, molecular docking is one of the most commonly used virtual screening approaches. It is possible to predict both the affinity of the ligand for a protein and the structure of the protein-ligand complex by using this technique, both of which are vital to lead optimization **(** Wang and Zhu, 2016)**.** Molecular docking aims to predict the structure of the ligand-receptor complex using computational methods. Docking is accomplished in two steps: sampling ligand conformations in the protein’s active site and then utilizing a scoring function to rate these conformations (Meng et al., 2012; Ferreira et al., 2015). A complete list of docking and ADME software are enlisted in Supplementary Table S9 (Meng et al., 2012; Singla et al., 2013; Ferreira et al., 2015; Dar and Mir, 2017; Kumar et al., 2018; Zhang et al., 2022) Supplementary Table S10 (Singla et al., 2013)**,** respectively.

### Discovery of covalent and noncovalent anti-tuberculosis drugs using molecular docking techniques

Covalent inhibition is a method for obtaining irreversible inhibition. Because covalent inhibitors may target proteins with shallow binding cleavage, new inhibitors with better efficacy than non-covalent inhibitors can be developed. Covalent molecular docking has recently been used in computer-aided drug design processes to characterize covalent interactions between inhibitors and biological targets. Several computational approaches for modeling covalent interactions have been developed. Autodock, Autodock Vina, GOLD, and FlexX are the most popular docking tools and software.

**FIGURE 8:**
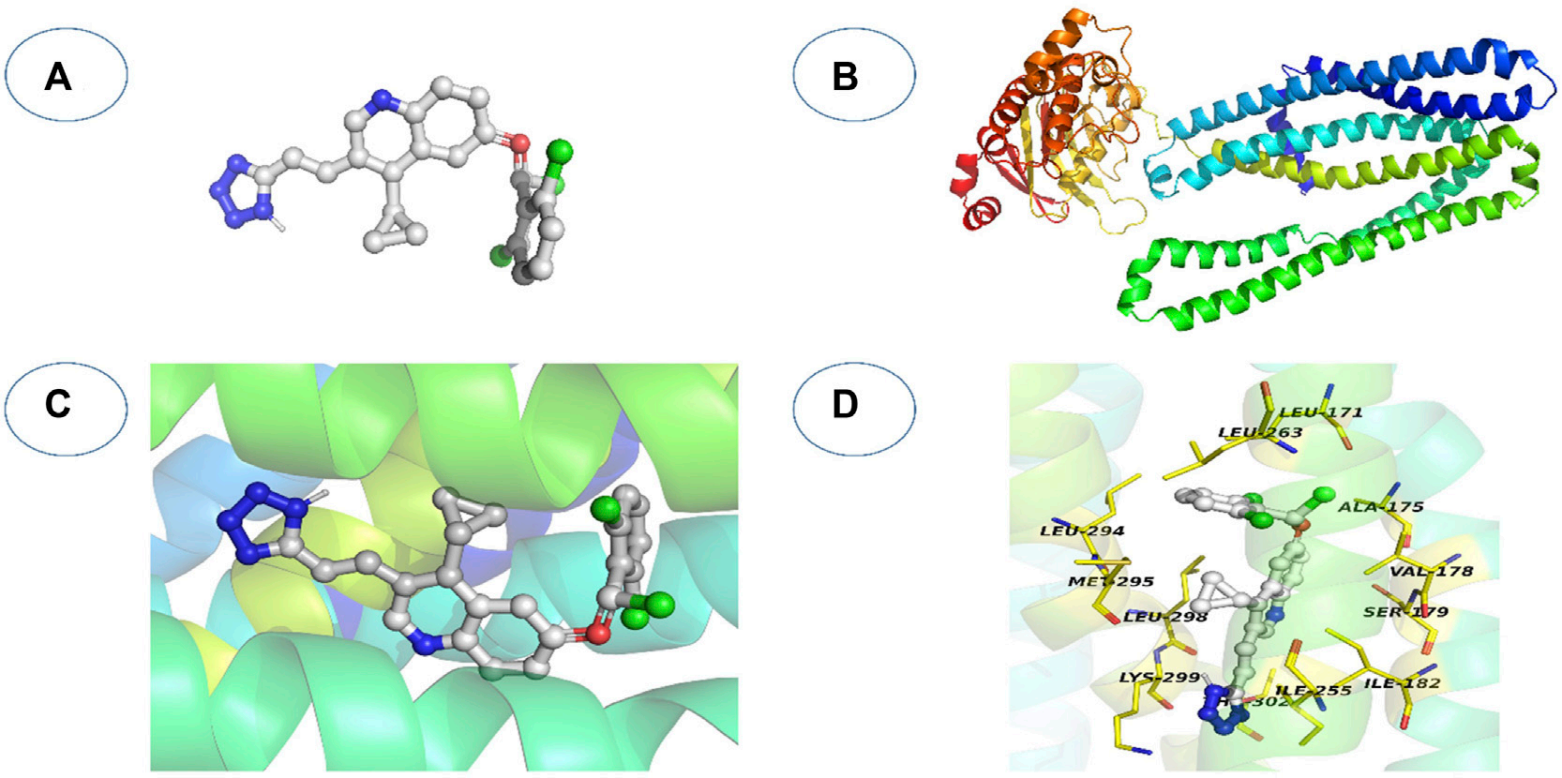
An overview of the docking process for molecules. **(A)** The ligand’s three-dimensional structure; **(B)** the 3D structure of the receptor; **(C)** The ligand is docked into the receptor’s binding cavity, and potential conformations are investigated.; **(D)** The detected intermolecular interactions and the most plausible binding conformation.

On the other hand, these and other similar methods primarily focus on non-covalent interactions (van der Waals interactions, electrostatic interactions, and hydrogen bonding) or the use of alternative empirical or knowledge-based scoring functions to characterize these non-covalent interactions **(** Kumalo et al., 2015)**.** Non-covalent interactions (van der Waals interaction, electrostatic interaction, and hydrogen bonding, for example,) or alternative empirical or knowledge-based scoring functions to characterize these non-covalent interactions are mainly used in these and other analogous methodologies. However, not all medications attach non-covalently to the active site; other compounds, such as covalent drugs (Singh et al., 2011), bind covalently. The workflow of covalent docking is represented in Figure 8 (Kumalo et al., 2015).

Many techniques have been developed to achieve the covalent docking of inhibitors to target proteins. However, most covalent docking methods can only predict the binding energy between an electrophilic ligand and a nucleophilic receptor. Popular is the “link atom” approach. The software defines a “link atom” in the ligand and the protein in this strategy. The ligand link atom must occupy the same steric volume as the protein link atom to mimic the covalent binding process. The Gold molecular docking program **(** Hartshorn et al., 2007) implements this strategy. Autodock, another widely used molecular docking program, uses a “grid-based technique” and a “modification of the flexible side chain” approach to covalently dock inhibitors to receptors. The covalently linked ligand and the protein attachment are sampled as part of the receptor as a single flexible side chain in a flexible side chain. A Gaussian biassing function focused on the protein attachment atom, and grid-based energy is used in the grid-based approach to bias the covalent bonding ligand posture **(** Kumalo et al., 2015)**.**

Katritch et al., 2007, used covalent docking and homology modeling to create a detailed structural model of the ubiquitin-like poxvirus proteinase (ULP) I7L substrate-binding site (S2–S2′). The 3D model of the I7L ligand-binding site was utilized to perform covalent docking and virtual screening of a comprehensive library of around 230,000 accessible ketone and aldehyde compounds to uncover novel smallpox antiviral hits. Out of 456 predicted ligands, 97 inhibitors of I7L proteinase activity were found to be active in biochemical studies (20 percent overall hit rate) **(** Katritch et al., 2007)**.** In their study, Wang et al. employed covalent and three-dimensional QSAR modeling to investigate the intermolecular interactions of isatin sulfonamide analogs as caspase-3 inhibitors (Wang and Zhu, 2016). Fifty-nine isatin sulfonamide analogs were docked to the binding site of human caspase-3. The docking research showed the inhibitors’ binding mechanism. A 3D-QSAR approach further supplements docking analysis by offering a “custom” scoring function for the protein under research, capable of predicting bioactivities for ligands comparable to those in the training sets. Structure-based design methodologies (such as docking) were shown to aid in constructing trustworthy QSAR models**.**

Ma et al. developed and covalently docked a novel family of peptide aldehyde derivatives to increase hydrophobic interactions with a bulky P3 moiety. Covalent docking was used to predict the exchange of the peptide aldehyde compounds with the 20S. The P3-position alterations are crucial for inhibitor efficacy. The docking behavior is similar to that of the crystal complex previously described. The hypothesized binding method might be leveraged to generate more potent 20S proteasome inhibitors **(** Ma et al., 2011)**.** A bicyclic class of covalent inhibitors was synthesized by Lawandi et al. to study the optimal shape required to target propyl oligopeptidase (POP) and heal human brain disorders. We hypothesized that these structures could bind covalently to the enzyme’s catalytic serine because they contain nitrile functional groups. From the covalent docking study, two compounds were chosen for production and biological testing. The study unveiled the presence of a potent, remarkably selective, and cell-permeable inhibitor for POP.

Furthermore, docking tests (using the FITTED docking engine and default parameters) demonstrated that the stereogenic center’s arrangement at the ring junction is a limiting factor for optimum activity, which may be used to guide future studies **(** Lawandi et al., 2009)**.** Saikia et al. study delve into the intriguing interaction between the antitubercular drug isoniazid (INH) and pristine and Si-doped and single-walled carbon nanotubes (SWNTs) (Saikia et al., 2016). The incorporation of silicon dopants significantly enhances the adsorption of INH onto the relatively inert nanotubes, as evidenced by profound alterations in adsorption energies, charge transfer phenomena, and global reactivity descriptor values. Notably, the study highlights noncovalent functionalization’s efficiency and mobility advantages over covalent counterparts, particularly in facilitating INH movement along the nanotube sidewall. Interestingly, parallel adsorption configurations manifest heightened charge transfer dynamics compared to the perpendicular adsorption orientation**.** The theoretical investigation posits single-wall carbon nanotubes as promising nano vectors for PZA (pyrazinamide) drug delivery, with covalent functionalization via sidewall and edge attachment, presenting a viable strategy. The study’s findings pave the way for further exploration, aiming to unravel the intricacies of therapeutic release from functionalized SWCNTs, ultimately enriching the landscape of future biomedical applications (Saikia et al., 2013)**.** The list of software used is given in Supplementary Table S11 (Singla et al., 2013).

### Post docking simulation studies for tuberculosis drug discovery

Molecular dynamics (MD) are more computationally demanding and sophisticated than simulations of other biomolecules. A collection of biomolecule conformations with distinct initial and boundary conditions can be generated by iteratively integrating (numerically) the equations of motion for specific potential simulations **(** Kumar et al., 2018)**.** A structural ensemble derived from an MD simulation is used to explore the conformational space of biomolecules, measure thermodynamic variables, and predict the free energy of biological processes. In calculations involving free energy binding, which encompasses a wide range of accuracies and processing needs, many people have tried the MD method to forecast the strength of non-bonded interactions. A very vital technique for the theoretical and computational studies of biomolecules is molecular dynamics simulation (MDS) **(** Huang et al., 2010)**.** Examining molecular systems is exceedingly tricky since they usually comprise many particles. Using numerical approaches in molecular dynamics simulation can avoid this analytical intractability. The atoms and molecules may briefly interact while the simulation is running. Each atom’s motion is calculated, and the overall behavior may be checked. It offers numerous advantages over docking because docking merely provides the ligand’s binding free energy among the receptor. MDS can also be used to predict the ligand’s actual interaction with receptors at the atomic level **(** Ferreira et al., 2015; Kumar et al., 2018).

Root means square deviation (RMSD) is applied in MDS to forecast receptor or ligand-receptor complexes for their stability and to explain conformational changes. An alternate conformational state consequently to such ligand-induced structure can be generated using MD simulations **(** Khan et al., 2016)**.** The projected ligand-receptor complex can be termed unstable if the A-matched docking solution and the ligand conformation produced by MD are different by more than a particular RMSD value **(** Kumar et al., 2018)**.** Coupled docking and MD approaches have been widely used to identify new therapeutic drugs from natural compounds and optimize the more recent lead candidate obtained from the natural compound **(** De Vivo et al., 2016)**.** MD is also applicable to produce a section of convenient docking structure, mainly when none of the acceptable crystallographic structures for the specific molecular target are offered **(** Harvey and De Fabritis, 2012)**.** MD simulations paired with molecular mechanics/Poisson-Boltzmann surface area (MM/PBSA) approaches can provide precise information on drug-target interaction binding effectiveness. This method has also been used to test the inhibitory efficacy of natural chemicals against various protein targets **(** Good, 2006)**.**

Using the MD simulation approach, various ways are available to examine atomic-level alterations in biomolecules (Khan et al., 2016 **).** Some, like Desmond, have a graphical user interface, while others, like GROMACS and AMBER, run via command lines. GROMACS (AMBER), Nanoscale MD (NAMD), and (CHARMM-GUI) are some well-known and commonly used MD simulation programs. The rise in software and hardware powers is crucial for executing such MD simulations **(** Kumar et al., 2018)**.**

It is a popular method for researching biologically known systems. It is also employed in various domains, including the Prediction of protein-ligand combination stability, protein mutation analysis, conformational protein stability prediction, and protein unfolding investigations. It is a powerful instrument demanding much computing power to solve biological puzzles **(** Hansoon et al., 2002; Hospital et al., 2015)**.**

### Molecules discovered by QSAR, pharmacophore modeling, and molecular docking for tuberculosis

A QSAR study was conducted on thirty-four 8-methyl quinolones by Eric et al. to determine their anti-tuberculosis activity. Picking descriptors and building association models relating to structural characteristics of biological activity was done using the genetic algorithm (GA) and multiple linear regression analysis (MLRA). The 3D structure of all compounds was constructed using the SPARTAN “14 v 1.1.0 tool. PADEL software for calculating molecular descriptors of all 34 compounds. The Build QSAR application was used to analyze geometric algorithms and build QSAR models. The internal validation was performed using the cross-validation leave-one-out technique, and external validation was performed by utilizing them to forecast the action of test sets. An examination of the robustness of the QSAR design was also conducted. The GA-MLRA method was used to construct a robust QSAR model to predict the inhibitory action of some quinolones. The results of this study (*R* ^2^ Pred = 0.7393) imply that for novel 8-methyl quinoline analogs, the pMIC can be calculated using the QSAR model that comes under the model’s applicability domain before synthesizing them (Eric et al., 2016)**.** It applied various computational methodologies to the 2,4-diamino quinazoline moiety to assess its efficacy in tuberculosis between diverse biological functions (Bose et al., 2019). V-Life MDS software was used to study all QSAR studies. Merck molecular force field was used to get 3D structures from 2D QSAR. In 3D QSAR, conformers were created using the conformational Monte Carlo technique, and the conformers with the lowest energy were chosen to align. The pharmacophore identification investigations were performed in the V-life MDS 4.4 Mol sign module to associate geometrical representation of the properties required for the molecule to show activation. The docking investigation was conducted using GOLD software. Thirty-three molecules were docked with four distinct protein molecules, significant for anti-TB action and crystalline protein structure. The new molecular set was designed depending on the pharmacophore results of docking studies and QSAR. After designing, the new molecule set was optimized, and using Monte Carlo conformation search conformers were generated. The 2,4-diamino quinazolines scaffold is promising for development as a molecular lead set for lead optimization, according to the findings of this study. According to the QSAR study, better moieties require a perfect distribution of steric potential and hydrophobicity in the molecular system. Pharmacophore mapping depends on the quinazoline ring’s 1 and 3 nitrogen atoms, the 4-amino phenolates molecule at location 4, the benzene ring, and the electronegative fluorine replacement. According to QSAR and pharmacophore mapping data, replacing the piperidine ring at the 2-amino position and the 4-amino phenolate group over the 4-amino position is the main necessity, as evidenced by the design and assessment of the activity using a 3D QSAR model. All of the features assessed and assumed to represent an active moiety were included in the final molecule **(** Bose et al., 2019)**.** Bhardwaj *et al.* explored the binding affinity of 70 novel piperine analogs and reported the activity of 23 compounds against *Mycobacterium tuberculosis*. The antitubercular effects of verapamil analogs, which are derivatives of dimethoxy phenyl rings bound to carbon chains, are significant. However, piperine, which also has a methylenedioxyphenyl ring attached to a carbon chain, is found to be a structural requirement for its activity. The similar action of these two compounds was correlated using *in silico* approaches. Using DRAGON and Chem office software, descriptors were calculated in QSAR studies. The QSAR models were generated by performing MLRA (Multiple Linear Regression Analysis) analysis using CODESSA^®^. The structures of the compounds were sketched and optimized using Chem Sketch software. Schrodinger, 2016–1’s GLIDE module investigated drug-receptor interactions and designed new compounds. www.rcsb.org provided the protein structure (3C3W), or the Protein Prep Wizard module was used to make the protein. The binding cavity within the protein was determined using site mapping, which will be utilized in future studies. Using a 2D sketcher, the structure of the ligands was created and optimized. For energy optimization, PLS3 was employed. It was concluded from QSAR and molecular docking studies that the binding energies range of predicted products with one hydrogen bond to five hydrogen bonds was higher than reported compounds with one hydrogen bond to four hydrogen bonds. Compared to reference medications, some predicted molecules have shown good binding affinity. As the docking scores are comparable, we may conclude that the QSAR models created are good, and these are applied for forecasting the anti-TB activity of novel drugs **(** Bhardwaj and Dubey, 2017)**.** The findings from the study underscore the power of integrating diverse computational strategies in the pursuit of anti-tubercular drug discovery. The combination of molecular docking, DFT calculations, reactivity descriptors, QSAR modeling, ADMET evaluation, and molecular dynamics simulations presents a formidable approach to expedite the identification of promising drug candidates (Stanzione et al., 2021). Ultimately, these advances contribute significantly to the ongoing battle against TB, offering hope for developing more effective treatments in the near future (Rajkhowa and Deka, 2013; Rajkhowa et al., 2015). A list of products discovered for tuberculosis is presented in Table 1. A list of the drugs (Table 2) in a clinical trial is discovered with the help of these approaches (Ahamad et al., 2017).

**TABLE 1:** List of some compounds discovered to target tuberculosis bacteria by using approaches of QSAR, pharmacophore modeling, and molecular docking.

Compound	Database	QSAR Method	Descriptor calculation	Method validation	Pharmacophore modeling	Molecular docking software	Result	Ref
Xanthone derivatives as Anti TB agent	Protein data bank	Multiple linear regression (MLR) backward methods		Parameterized Model Number 3 (PM3), Austin Model 1 (AM1), and Density Functional Theory (DFT), Hartree-Fock (HF)	--	CHIMERA 1.9 and ChemOffice^®^2015	The 3,6 dihydroxy and 1,3,6 trihydroxy xanthone derivatives have good anti-tuberculosis activity when added to amide, sulfoxide, and carboxylate groups. A docking study was used to identify the inhibitory mechanism known as Kasa inhibitor, which is located in the cell wall of *Mycobacterium* TB.	Yuanita et al. (2020)
Sulfathiazole Analogs such as *mycobacterium tuberculosis* h37rv Inhabitors	Antituberculosis drug discovery databases (Substructure mining tool Schrodinger et al., 2010)	Principal Component Regression (PCR) Analysis, Multiple linear regression (MLR), Partial Least Squares (PLS) Regression Analysis	Vlife MDS	External validation by predicting the activity of each molecule in the test set Internal validation (Leave-one-out)	--	--	Compared to the other two methods in predicting the antituberculosis H37RV inhibitor effect of sulfathiazole analogs, PLS analysis demonstrated significant predictive power and reliability	Vastrad (2012)
Amino-pyrimidine derivatives as *Mycobacterium tuberculosis* Protein Kinase B inhibitors	Literature based on the biological assay method	MLR	--	Internal and external validation	--	--	With an excellent statistical fit, the QSAR model was created using MLR. Antituberculosis action was discovered to be influenced by physicochemical molecular and quantum descriptors. The researchers concluded that a model like this might be used to predict the antituberculosis action of these compounds	Chapman et al. (2012)
The pharmacophore model was used as a tool to identify a novel inhibitor of Mtb-DapB, a validated mycobacterial drug target	The ZINC natural product subsets and Asinex screening library	X-ray Crystal structure 1C3V	--	--	e-Pharmacophore option from the Phase module of the Schrodinger Suite		It was discovered that hybrid dynamic pharmacophore models created by employing a computation-based technique to screen compounds for new chemotypes, higher binding affinities, and drug-like features outperformed traditional models made from native ligands. Based on cheminformatics-based structure comparison, docking scores, binding energies, and ADMET properties, the compounds screened by the hybrid pharmacophore models were discovered to be more druglike, defining the hybrid models as useful tools for exploring novel anti-TB chemical space	Choudhury and Bhardwaj, (2020)
Predicting the activity of 1,2,3-triazole and pyrazolopyridones as DprE1 inhibitor antitubercular agents	Literature	MR, Principal PCR, PLSR and PLS-SE) the method used to develop 4 QSAR models	V-life MDS	Internal and external validation	The MolSign module in VLifeMDS	The Biopredicta tool of V-Life MDS software version 4.6	Utilizing pharmacophore modeling, QSAR analysis, molecular docking, and *in silico* ADME prediction, the function of 1,2,3-triazole and pyrazolopyridones as DprE1 inhibitors antitubercular drugs were explored, offering input into the structural foundation and inhibitory mechanism represents the group of substances serving as DprE1 antitubercular agents	Panigrahi et al. (2020)
Quinoline Schiff bases as enoyl acyl carrier protein reductase inhibitors	Literature	CoMFA, CoMSIA, and topomer CoMFA 3D structure of quinoline scaffold using molecular modeling software package SYBYL-X 2.0	--	--	--	Surface docking	The study proved that the presence of the -CH = N- and quinoline rings are critical for anti-TB activity. It was also discovered that compounds had a higher lipophilic character, making them capable of demonstrating positive biological activities. The reported models could be further investigated to develop newer, more powerful anti-TB drugs	Joshi et al. (2014)
QSAR and docking studies of pyrimidine derivatives against *M. tuberculosis* H37Rv	--	MLR, Stepwise selection of Terms (SW)	--	--	--	AutoDock	The study’s findings suggested that modifying and substituting the pyrimidine ring could result in a possible lead chemical with antibacterial activity and good docking. The findings of QSAR and docking studies on pyrimidine derivatives will aid the introduction of innovative antituberculosis medications	Hussain et al. (2016)
QSAR-driven Design, Synthesis, and Discovery of Potent Chalcone Derivatives with Antitubercular Activity	Bioassay, PubChem, SciFinder database, ChEMBL, also from literature	Avalon fingerprints, combined with support vector machine (SVM) gradient boosting machine (GBM), and random forest (RF) machine learning methods, MACCS, AtomPair, Morgan, FeatMorgan	--	--	--	--	Identifying novel and promising anti-TB drugs were made possible by integrating into silico design a QSAR-driven pathway for screening, production, and experimental evaluation. Thirty-three chalcone derivatives were created and evaluated against *Mycobacterium tuberculosis* strains. The synthesized chalcone compounds were proven effective against mono-resistant M. TB strains of isoniazid and rifampicin. The compounds were not harmful to mammalian (VERO) cells and appeared to be mycobacteria-specific, with just a little effect on *S. aureus*	Gomes et al. (2017)

**TABLE 2:** A list of the drugs that are in a clinical trial is discovered with the help of these approaches.

Sr No.	Drug	Target	Clinical trial stage
1	Bedaquiline	ATP synthase	Phase 2
2	SQ109	Cell wall synthesis (MmpL3)	Phase 2
3	Gatifloxacin	DNA gyrase	Phase 4
4	AZD5847	Reducing the ribosome’s initiation step	Phase 1
5	Linezolid	Ribosome	Phase 2
6	Pretomanid	Cell wall inhibition	Phase 1
7	Sutezolid	Ribosome	Phase 2
8	Moxifloxacin	DNA gyrase	Phase 2
9	Clofazimine	Electrogenic pathway	Phase 2

### The challenges and prospects of QSAR, pharmacophore modeling, and docking of molecules

#### QSAR challenges and prospects

The chemical descriptors used as input for the QSAR model vary throughout most QSAR research. The quality to predict traditional QSAR approaches reduces when new substituent group traits depart further from the training set. Some of the challenges associated with 3D QSAR are ligand geometry which is essential for computing geometric descriptors and crucial for 3D QSAR. QSAR data sets may contain many chemicals (>100,000) and descriptors. There is a requirement to maintain many models (e.g., dozens) for a variety of targets; these models should be updated regularly (e.g., monthly) (Winkler, 2002; Cherkasov et al., 2014; Saikia and Bordoloi, 2019)**.** Failure to take data heterogeneity into account, unsuitable endpoint units used, confounded and non-interpretable descriptors being used, descriptor value errors, QSARs having a low transferability, applicability domain is insufficient or undefined, omission of data points that are unnoticed, data that is insufficient, chemicals in a data set are replicated, endpoint values within a narrow range, data that has been over-fitted, in a QSAR, using an excessive number of descriptors, Statistics that are insufficient or missing are also misused and misrepresented, incorrect calculation, the complexity of the data set, validation of QSAR models, establishing the models’ scope of application in the chemical space **(** Aparoy et al., 2012; Ma et al., 2015)

Significant-scale QSAR research is possible due to large data and processing resources. Graphic processing units, Cloud technology, and servers are very known and have been used in computer-aided drug design and discovery streams **(** Aleksandrov and Myllykallio, 2019)**.** The system pharmacology method has gained popularity among scientists due to its ability to conduct pharmacodynamic assessments, discover new targets, and provide a systems-level understanding of drug-disease interactions **(** Maltarollo et al., 2017)**.** Combining QSAR with machine learning methods enhances prediction power and is needed for the future development of QSAR **(** Sabitov et al., 2017)**.**

#### Pharmacophore modeling: Challenges and prospects

The pharmacophore approach still faces numerous roadblocks that limit its ability to reach its full potential, especially given the current high Costs involved in medication discovery and development. The following are some of the challenges: The challenge associated with ligand-based pharmacophore modeling is the modeling of ligand flexibility. The second difficulty encountered when adopting the ligand-based strategy is molecular alignment. Screening massive chemical databases with flexible compounds, a fundamental problem in pharmacophore-based VS., could take quite a long time. In many cases, the most challenging issue with pharmacophore-based VS. is that a minor quantity of simulated hits is genuinely bioactive **(** Leach et al., 2010; Qing et al., 2014)**.** The absence of a good scoring function in virtual screening by pharmacophore is also one of the challenges faced while using pharmacophore-based virtual screening. A pre-computed conformation database is necessary for a pharmaceutical-based virtual screening which is also a challenge in its application **(** Qing et al., 2014)**.** The *de novo* design using the pharmacophore-based program new LEAD has the limitation that new LEAD can only handle pharmacophore properties that are concrete functional groups rather than abstract chemical features (Leach et al., 2010; Yang, 2010)**.** The correct selection of the training set molecules is also a difficult task. Another drawback is the lack of a clear approach to generating a pharmacophore query. Finding different conformations for each ligand is also challenging using the pharmacophore model. Another significant drawback is that the pharmacophore confirmation may not be a ligand active form based on free energy. Every ligand-based pharmacophore carries this risk (Seidel et al., 2020; Muhammed and Aki, 2021).

A combination of pharmacophore modeling and other computational techniques is needed to overcome some of the limitations of pharmacophore modeling and stay on top of recent discoveries **(** Qing et al., 2014)**.** As a result, pharmacophore modeling has been combined using molecular mechanics simulations. This might help to increase some of the difficulties associated with ligand flexibility modeling. Another issue noted is the absence of good scoring functions utilized in virtual screening by pharmacophores. Machine learning, which has been utilized in various computational ways, can be used to improve such scoring functions. As a result of recent improvements in pharmacophore modeling, structure-based models can now be produced with enhanced properties (Yang, 2010; Kumar et al., 2018; Muhammed and Aki-Yalcin, 2021)**.**

#### The challenges and future of molecular docking

The market for drug discovery informatics is expected to rise from 1.5 billion dollars in 2016 to 2.84 billion dollars in 2022, and it may continue to grow. As a result, there is a growing demand for innovative informatics solutions development and implementation. Moving from pure research to clinical therapy is one of the primary drivers driving the growth of the worldwide market. More qualified individuals, multidisciplinary backgrounds, and the high cost of informatics software might significantly influence the market’s growth. Several well-known programs have recently been made accessible as free or paid software or services. It will take a considerable amount of time, effort, and resources to fully exploit the potential of this robust approach (Meng et al., 2012; Akhter, 2016; Wang and Zhu, 2016; Dar and Mir, 2017; Kumar et al., 2018; Lambrinidis and Tsantli, 2018; Prieto-Martínez et al., 2018).

Here are some of the difficulties that come with molecular docking. Choosing optimal methods, tools, and parameters in molecular docking is difficult **(** Naveja and Medina, 2019). Molecular docking methods generally neglect solvent influence, entropic effects, and polarization for binding ligands. Experimental scoring formulas have been developed to overcome such obstacles **(** Ganesan, 2008; Benet et al., 2016)**.** Receptor flexibility is a significant challenge in molecular docking. Docking had another issue with the scoring function’s imperfection. When docking molecules, keeping track of their numerous tautomeric and protomeric states can be challenging (Benet et al., 2016)**.** Evaluating molecular docking results without considering the receptor structure’s quality is also difficult. Protein flexibility is also an important point to be considered, as ligand binding to protein produces conformation changes in protein, hence ignoring protein flexibility may produce incorrect results during molecular docking. Handling flexible protein receptors is a major challenge during molecular docking **(** Eldridge et al., 1997; Allouche, 2012)**.**

The development of Local Move Monte Carlo (LMMC) based molecular docking in the future, where A sample that considers the backend loops in addition to the main chain in the ligand binding of proteins and flexible ligands may be a workable solution to the flexible receptor docking issue. Docking applications could be propelled to the next level by accurate and low-cost scoring techniques **(** Ganesan, 2008; Benet et al., 2016)**.** For continued progress, the molecular structure databases must be improved. Filters must ensure that the structural models inside them are of higher quality, as this will affect the findings’ dependability. In 1971 the protein data bank (PDB) was created as a pioneer crystal structure database. It is now the most widely used molecular *in silico* modeling resource, with over 150,000 experimentally validated 3D models. However, even with acceptable geometrical parameters, there is no assurance that the selected structures are error-free, which must be considered. The presence of high-quality statistics does not imply that the structure is flawless. As a result, improving their quality, procedures, and validation would allow better models to be built, which would be helpful in the inevitable process of structural refinement.

On the other hand, a better model will not be more instructive in terms of more complex biological information, necessitating a scientist’s interpretation **(** Lambrinidis and Tsantli, 2018)**.** Despite this, the accuracy of the docking tool and the validity of the results can be evaluated. Although docking procedures have gotten more complicated, false positives are still a problem with this methodology; refining the PDB structures would surely enhance pharmacodynamics research and yield better findings.

## Conclusion

QSAR models can be used during drug development research and development stages to develop pharmacodynamic and pharmacokinetic profiles. These *in silico* investigations predict numerous characteristics and actions that aid in optimizing and prioritizing drug molecules. Computational approaches have resulted in lead structures and novel drug targets, which have sped up drug development. The computational technique may find pharmacological leads and targets against them, as well as attraction and effectiveness between them, before the start of clinical trials; it saves time and money. The QSAR is a commonly used statistical method that links a molecule’s structure to its physiological action meant for a function of molecular descriptors. Hence, it plays a vital role in drug development. Pharmacophore is essential for a compound’s biological action. It aids in *de novo* design, important characteristics, and high-throughput screening in drug development. In addition to their many applications in the examination of specific molecular activities, structure-based virtual screening (SBVS) molecular dynamics (MD), bounding energy, molecular interactions, molecular docking, and structure-based virtual screening (SBVS) are some of the most popular strategies used in structure-based drug discovery (SBDD). The review article concluded that such techniques will reduce traditional resource requirements by increasing prediction based on current information and limiting and focusing on chemical production and biological testing.
