# Corrigendum: Navigating bioactivity space in anti-tubercular drug discovery through the deployment of advanced machine learning models and cheminformatics tools: a molecular modeling based retrospective study

**DOI:** 10.3389/fphar.2023.1340724

**Published:** 2024-01-09

**Authors:** Ratul Bhowmik, Ravi Kant, Ajay Manaithiya, Daman Saluja, Bharti Vyas, Ranajit Nath, Kamal A. Qureshi, Seppo Parkkila, Ashok Aspatwar

**Affiliations:** ^1^ Medicinal Chemistry and Molecular Modelling Lab, Department of Pharmaceutical Chemistry, School of Pharmaceutical Education and Research, New Delhi, Delhi, India; ^2^ Medical Biotechnology Laboratory, Dr. B. R. Ambedkar Center for Biomedical Research, University of Delhi, Delhi, India; ^3^ Department of Bioinformatics, School of Interdisciplinary Studies, Jamia Hamdard University, New Delhi, India; ^4^ Department of Pharmaceutics, School of Pharmaceutical Sciences, Siksha ‘O’ Anusandhan University, Bhubaneswar, Odisha, India; ^5^ Department of Pharmaceutics, Unaizah College of Pharmacy, Qassim University, Unaizah, Saudi Arabia; ^6^ Faculty of Medicine and Health Technology, Tampere University, Tampere, Pirkanmaa, Finland; ^7^ Fimlab Ltd., Tampere University Hospital, Tampere, Pirkanmaa, Finland

**Keywords:** molecular docking, tuberculosis, drug resistance, QSAR, pharmacophore modeling

In the published article, there was an error in the **Author** list, and author “Ravi Kant^2^ and Daman Saluja^2^” was erroneously “excluded”. The corrected **Author** list appears below.

“Ratul Bhowmik^1^, Ravi Kant^2^, Ajay Manaithiya^1 *^, Daman Saluja^2^, Bharti Vyas^3^, Ranajit Nath^4^, Kamal A. Qureshi^5^, Seppo Parkkila^6,7^, and Ashok Aspatwar^6^ *

1 Medicinal Chemistry and Molecular Modelling Lab, Department of Pharmaceutical Chemistry, School of Pharmaceutical Education and Research, Jamia Hamdard, New Delhi, India

2 Medical Biotechnology Laboratory, Dr. B. R. Ambedkar Center for Biomedical Research, Delhi School of Public Health, IoE, University of Delhi, Delhi, India

3 Department of Bioinformatics, School of Interdisciplinary Studies, Jamia Hamdard, New Delhi, India

4 Department of Pharmaceutics, School of Pharmaceutical Sciences, Siksha ‘O’ Anusandhan University, Bhubaneswar, Odisha, India

5 Department of Pharmaceutics, Unaizah College of Pharmacy, Qassim University, Unaizah, Al-Qassim, Saudi Arabia

6 Faculty of Medicine and Health Technology, Tampere University, Tampere, Finland

7 Fimlab Ltd., Tampere University Hospital, Tampere, Finland”

The authors apologize for this error and state that this does not change the scientific conclusions of the article in any way. The original article has been updated.

