# Supplementary material for: Navigating bioactivity space in anti-tubercular drug discovery through the deployment of advanced machine learning models and cheminformatics tools: a molecular modeling based retrospective study
Source: Front Pharmacol. 2023 Aug 29;14:1265573. doi: 10.3389/fphar.2023.1265573 (PMC10495588; doi:10.3389/fphar.2023.1265573)
Supplement: Supplementary file 1 [file DataSheet1.docx]

**Supplementary File**

**Navigating Bioactivity Space in Anti-Tubercular Drug Discovery through the Deployment of Advanced Machine Learning Models and Cheminformatics Tools: A molecular modeling based retrospective study**

Ratul Bhowmik**^1^**, Ajay Manaithiya**^1,*^**, Bharti Vyas**^2^**, Ranajit Nath**^3^,** Kamal A. Qureshi**^4^**, Seppo Parkkila**^5,6^**, Ashok Aspatwar**^5,*^**

^1^Medicinal Chemistry and Molecular Modelling Lab, Department of Pharmaceutical Chemistry, School of Pharmaceutical Education and Research, Jamia Hamdard, New Delhi-110062, India

^1,2^School of Interdisciplinary Studies, Jamia Hamdard, New Delhi-110062, India

^3^Siksha 'O' Anusandhan University, Bhubaneswar, Odisha-751030, India

^4^Department of Pharmaceutics, Unaizah College of Pharmacy, Qassim University, Unaizah, Al-Qassim-51911, Saudi Arabia

^5^Faculty of Medicine and Health Technology, Tampere University, Tampere-33520, Finland

^6^Fimlab Ltd., Tampere University Hospital, Tampere-33520, Finland

**Table 1.** List of webservers or databases for QSAR drug design research

| **Sr. No.** | **Database** | **Brief Description with URL** |
| --- | --- | --- |
|  | Aureus Pharma | Chemical structure, biological activities (*in vitro, in vivo*) <http://www.aureus-pharma.com> |
|  | ChemBank  ChemIDPlus | Chemical structure, toxicity,  Physicochemical properties, etc.  <http://chembank.broad.harvard.edu>  <http://chem.sis.nlm.nih.gov/chemidplus> |
|  | Chemoinformatics.org | Data sets  <http://www.cheminformatics.org> |
|  | ChemSpider | Physicochemical properties  <http://www.chemspider.com> |
|  | Developmental therapeutics  program (DTP) | Chemical structure (2D and 3D),  biological data (oncology)  <http://dtp.nci.nih.gov/webdata.html> |
|  | QSAR World | Literature, data sets  <http://www.qsarworld.com> |
|  | SciFinder (CAS  Database) | Literature, chemical structure  <http://www.cas.org/> |
|  | PubChem | database of bioassays, compounds, and substances <http://pubchem.ncbi.nlm.nih.gov/> |
|  | ChEMBL | Database of drug-like molecules  <https://www.ebi.ac.uk/chembldb> |
|  | Zinc | Maintain commercially-available compounds for virtual screening  <http://zinc.docking.org/> |
|  | ChemDB | Collection of small-molecules  <http://cdb.ics.uci.edu/> |
|  | MMsINC | Commercial compounds  <http://mms.dsfarm.unipd.it/MMsINC/> |
|  | KEGG | Maintain comprehensive information  <http://www.genome.jp/kegg/> |
|  | SMPDB | Small molecule Pathway database  <http://www.smpdb.ca> |
|  | PDBeChem | Dictionary of chemical components referred to in PDB entries  <http://www.ebi.ac.uk/pdbe-srv/pdbechem/> |
|  | PDB-Bind | Binding affinity information for PDB Ligands  <http://sw16.im.med.umich.edu/databases/pdbbind/index.jsp> |
|  | BindingDB | The binding affinity of PDB Ligands  <http://www.bindingdb.org/> |
|  | NCI | Small molecules related to cancer  <http://cactus.nci.nih.gov/ncidb2.1/> |
|  | CDD | Collaborative drug discovery  <https://www.collaborativedrug.com/> |
|  | DrugBank | All kinds of drugs  [http://www.drugbank.ca](http://www.drugbank.ca/) |
|  | HMRbase | Hormones and their Receptors  <http://crdd.osdd.net/raghava/hmrbase/> |
|  | BIAdb | Benzyl-isoquinaloid alkaloids  <http://crdd.osdd.net/raghava/biadb/> |
|  | NPACT | Plant-derived natural compounds  <http://crdd.osdd.net/raghava/npact/> |
|  | Supernatural | A searchable database of available natural compounds |
|  | HIT | Herb ingredients target  <http://lifecenter.sgst.cn/hit/> |
|  | Drugs@FDA | drug products approved by FDA  <http://www.fda.gov/Drugs/> |

**Table 2.** List of software used in QSAR for file conversion or structure drawing

| **Sr. No.** | **Software** | **Brief description** |
| --- | --- | --- |
|  | ChemDraw | For chemical structure drawing and editing  <http://www.cambridgesoft.com/software/ChemDraw/> |
|  | ACD/ChemSketch | To draw chemical structures including organics, organometallics, polymers, and Markush structures  <http://www.acdlabs.com/resources/freeware/chemsketch/> |
|  | Open Babel software | Software for file conversion  <http://openbabel.org/> |

**Table 3.** List of Software used in QSAR for 3D structure generation

| **Sr. No.** | **Software** | **Brief description** |
| --- | --- | --- |
|  | CORINA | Used for generating 3D structures of small- and medium-sized compounds  <http://wwwmolecular-networks.com/products/corina> |
|  | Concord | It is used to convert 2D inputs to 3D structures  <http://www.tripos.com/index.php?family=modules,SimplePage...&page=sybyl_conord> |
|  | Frog | It is an online tool that is used to generate 3D confirmation from 1D or 2D data utilizing the Merck molecular force field  <http://bioserv.rpbs.jussieu.fr/Help/Frog-Help.html> |
|  | Smi23d | To generate 3D structures  <http://www.chembiogrid.org/cheminfo/smi23d/> |

**Table 4.** Classification of 3D-QSAR approaches

| **Sr. No.** | **Classification** | **Example** |
| --- | --- | --- |
|  | **Based on intermolecular modeling, or the information used to develop QSAR** | |
|  | Based on receptors 3D-QSAR | COMBINE, AFMoC, HIFA, CoRIA |
|  | Based on ligands 3D-QSAR | CoMFA, CoMSIA, COMPASS, GERM, CoMMA, SoMFA |
|  | **Based on the alignment criteria** | |
|  | Alignment-dependent 3D-QSAR | COMPASS, CoMMA, HQSAR, WHIM, EVA/CoSA, GRIND |
|  | Alignment-independent 3D-QSAR | CoMFA, CoMSIA, GERM, COMBINE, AFMoC, HIFA, CoRIA |
|  | **Based on the method of chemometric correlation between structural characteristics and activities** | |
|  | Non-linear 3D-QSAR | COMPASS, QPLS |
|  | Linear 3D-QSAR | CoMFA, CoMSIA, AFMoC, GERM, CoMMA, SoMFA |

**Table 5.** list of some of the QSAR descriptors

| **Sr. No.** | **Year** | **Index** |
| --- | --- | --- |
|  | **Topological (2D) descriptors** | |
|  | 1947 | Wiener |
|  | 1972 | Zagreb indices |
|  | 1985 | Kappa shape |
|  | 1993 | Hyper-Wiener index WW |
|  | 2001 | Modified Hosoya index Z* |
|  | **Geometric (3D) descriptors** | |
|  | 1977 | Using electron diffraction, 3D-molecular representations of structures are possible (MoRSE) |
|  | 1995 | 3D Autocorrelation |
|  | 2002 | Geometry, Topology, and Atom-Weights AssemblY (GETAWAY) |

**Table 6.** List of software used for calculating descriptors and fingerprints

| **Sr. No.** | **Software** | **Brief Description** |
| --- | --- | --- |
|  | Joelib | Descriptor calculation software  <http://sourceforge.net/projects/joelib> |
|  | Afgen | Fragment-based descriptors  <http://glaros.dtc.umn.edu/gkhome/afgen/overview> |
|  | ISIDAfragmentor | Calculate Substructural Molecular Fragments and ISIDA Fragments  <http://infochim.u-strasbg.fr/spip.php?rubrique49> |
|  | ODDesripotrs | Simple java-based command level tool for descriptor calculation [http://www.softpedia.com/get/Science- CAD/ODDescriptors.shtml](http://www.softpedia.com/get/Science-%20CAD/ODDescriptors.shtml) |
|  | MOLD2 | Calculating descriptors from a two-dimensional chemical structure  (<http://www.fda.gov/ScienceResearch/BioinformaticsTools/Mold2/default.htm> |
|  | PowerMV | Window-based calculation of descriptors  <http://nisla05.niss.org/PowerMV/index.html> |
|  | PaDEL | Fingerprints calculation  <http://padel.nus.edu.sg/software/padeldescriptor> |
|  | CDK | Chemistry Development Kit  <http://cdk.sourceforge.net> |
|  | MODEL | A web server for molecular descriptors based on the 3D structure  <http://jing.cz3.nus.edu.sg/cgi-bin/model/model.cgi> |
|  | ChemDes | Molecular descriptors  [www.scbdd.com/chemdes](http://www.scbdd.com/chemdes) |
|  | E-DRAGON | Molecular descriptors  [www.vcclab.org/lab/edragon/](http://www.vcclab.org/lab/edragon/) |
|  | Molconn-Z | Topological descriptors  [www.edusoft-lc.com/molconn](http://www.edusoft-lc.com/molconn) |

**Table 7.** Software of machine learning and algorithms for developing QSAR model

| **Sr. No.** | **Software** | **Website** |
| --- | --- | --- |
|  | SVM | <http://www.cs.cornell.edu/People/tj/svm_light/> |
|  | ANN | <http://www.ra.cs.uni-tuebingen.de/SNNS/> |
|  | **Algorithms** | |
|  | JCsearch | <http://www.chemaxon.com/jchem/doc/user/Jcsearch.html> |
|  | PubChem | <http://pubchem.ncbi.nlm.nih.gov/search/> |
|  | SUBCOMP | <http://www.genome.jp/tools/subcomp/> |

**Table 8.** Different types of softwares and algorithms used for pharmacophore model generation

| **Sr. No.** | **Software** | **Website** |
| --- | --- | --- |
|  | PHASE | Pharmacophore modeling  <http://www.schrodinger.com/Phase/> |
|  | Catalyst | It uses two algorithms, Hypogen and HipHop algorithms  <http://accelrys.com/products/discovery-studio/pharmacophore.html> |
|  | Align-it  (Pharao) | Pharmacophore alignment  <http://silicos-it.be> |
|  | FLAP | Fingerprints using pharmacophoric features  <http://www.moldiscovery.com/software/flap/> |
|  | LigandScout | Pharmacophore modeling  <http://www.inteligand.com/ligandscout/> |
|  | MOE | Pharmacophore modeling  <http://www.chemcomp.com/MOE-Pharmacophore_Discovery.html> |
|  | MolSign | Pharmacophore modeling  <http://www.vlifesciences.com/products/Functional_products/Molsign.php> |
|  | PharmaGist | Pharmacophore detection  <http://bioinfo3d.cs.tau.ac.il/pharma/> |
|  | Pharmer | Pharmacophore search  <http://smoothdock.ccbb.pitt.edu/pharmer/> |
|  | PharmMapper | Drug target identification  <http://59.78.96.61/pharmmapper> |
|  | Quasi | Pharmacophore modeling  <http://www.certara.com/products/molmod/sybyl-x/simpharm/> |

**Table 9.** List of software for Molecular docking conformational search techniques and scoring functions

| **Sr. No.** | **Systematic Search** | **Random/Stochastic Search** |
| --- | --- | --- |
|  | eHiTS | AutoDock  http://autodock.scripps.edu/ |
|  | FRED | Gold  http://www.ccdc.cam.ac.uk/ |
|  | Surflex-Dock | PRO_LEADS |
|  | DOCK  http://dock.compbio.ucsf.edu/ | EADock |
|  | GLIDE  http://www.schrodinger.com/ | LigandFit  http://www.3dbiovia.com/ |
|  | EUDOC | Molegro Virtual Docker |
|  | FlexX (http://www.biosolveit.de/products/) | CDocker |
|  | SLIDE | AutoDock Vina - enhanced AutoDock 4; it is quick and has a better binding affinity. (http://vina.scripps.edu/) |

**Table 10.** List of software for ADMET properties prediction

| **Sr. No.** | **Systematic Search** | **Random/Stochastic Search** |
| --- | --- | --- |
|  | OSIRIS | ADMET <http://www.organic-chemistry.org/prog/peo/> |
|  | Metabolizer | Drug metabolism  <http://www.chemaxon.com/products/onlinetryouts/metabolizer/> |
|  | ToxTree | Toxicity estimation  <http://toxtree.sourceforge.net/download.html#Toxtree_2.5.0> |
|  | admetSAR | ADMET prediction  <http://www.admetexp.org/predict/> |
|  | FAF-Drugs2 | ADMET  <http://www.mti.univ-parisdiderot.fr/fr/downloads.html> |
|  | MetaSite | Metabolism site prediction  <http://www.farma.ku.dk/smartcyp/download.php> |
|  | MetaPred | Prediction of metabolism of Compound  <http://crdd.osdd.net/raghava/metapred/> |

**Table 11.** List of software for covalent and non-covalent docking

| **Sr. No.** | **Software** |
| --- | --- |
|  | AutoDock4 |
|  | CovDock |
|  | FITTED |
|  | GOLD |
|  | ICM-Pro |
|  | MOE |
|  | FlexX |

**2.3 Analyses of exploratory data on QSAR models**

Learning is the process of obtaining knowledge through study and prior experience. The types of learning in multivariate analysis are supervised and unsupervised learning. On data sets with known dependent variables, supervised learning is used. The algorithm can modify its performance following the error produced since the data set contains the values of dependent variables, which is common in the back-propagation neural network **[43].** Unsupervised learning categorizes or predicts the class to which a molecule belongs without using dependent factors. Due to the presence of dependent variables in supervised learning data sets, training, and test sets are necessary for evaluating prediction performance. As a result, in an unsupervised learning approach like SOM, all molecules in the data set are utilized to create a map that shows how comparable molecules are grouped.

In contrast, unrelated molecules are spread out further **[31].** Exploratory data analysis (EDA) and unsupervised pattern recognition are two popular ways to make data structures more straightforward. PCA is the most often used EDA method. Other unsupervised pattern recognition approaches, such as cluster analysis (CA), can be used to quickly assess the information richness of data tables **[44].**

- - 1. **Principal Component Analysis (PCA)**

Rather than developing a QSAR model, it is another data reduction strategy that looks for correlations between independent variables. It then creates a new set of orthogonal descriptors known as principal components (PCs), which describe most of the information in the independent variables in decreasing order of variance **[13]**. As a result, PCA reduces the dimensionality of a multivariate data collection of descriptors to the data available at the time. The principal components regression approach is utilized when principal components are used as independent variables in linear regression (PCR). In other words, PCR uses the PCA decomposition scores as regressors in the QSAR model to create a multiple-term linear equation **[13,21,30].** The primary components (PCs) are discovered in the core of a data stream where the variance is highest. Typically, a computer consists of two parts: data structures and noise. The data structure contains the basic information about the descriptor matrix. Data in the descriptor matrix unrelated to the response variables are called "noise." The early PCs preserve the data set's data structures, as indicated by the considerably explained variation. Later computers, on the other hand, are made entirely of noise components. The PCs are small, orthogonal, and operate as predictors of response variables. Because PCs are nonlinear, duplicate variables do not affect them. Consequently, PCs may be used to choose features quickly **[43, 45].**

PCA must subtract the mean from each data dimension to function correctly. Apart from their distinct nomenclature, the three most often used numerical algorithms for PCA decomposition are Eigenvector–eigenvalue extraction, PCA, and singular value decomposition (SVD). The PCA decomposition can be expressed as follows for any centered data matrix X (m, n), corresponding to m samples and n descriptors **[44].** The following equation (1) contains:

**X= TPT…………………. eq.1**

The matrix T (m, r) indicates the location of the compounds in the new coordinate system generated by PCs in the axis and contains the data scores. P is the dimension (n, r) loading matrix, where r represents the mathematical rank of the data, which is equal to min (m, n). The generation of PCs from original variables is described in the P columns (old axes). Loading is the estimation of the information shared between a component and a variable. Significant components indicate data compression degrees and are usually much bigger than the number of original variables, n. The compression of the examined data set will be better with a higher degree of correlation among the original variables, resulting in fewer significant principal components. The number of substantial PCs can be determined using cross-validation processes such as the bootstrap and the jackknife [46]. PCA is based on the eigendecomposition of positive semi-definite matrices and singular value decomposition (SVD) of rectangular matrices in mathematics **[45].**

Outliers are particularly sensitive to PCA, as they are to any least square technique. Outliers can affect model error by changing the direction of major components **[47].** The robust PCA approaches are designed to find principal components that aren't significantly influenced by outliers. Many methods have been proposed to accomplish this goal. These approaches are typically divided into three categories: i) approach that takes a set of resilient eigenvectors and eigenvalues utilizing a robust covariance matrix. (ii) projection pursuit methods that directly yield robust estimates of eigenvectors and eigenvalues without requiring the robust estimate of the covariance matrix, and (iii) a hybrid of both **[48,34].**

There have been several projection pursuits (PP) approaches presented. The critical distinction between them is the projection index employed. ROBPCA is a typical robust PCA technique that combines projection pursuit with robust covariance estimation in a low-dimensional environment. This method is also well adapted to high-dimensional data analysis and may be applied to a range of multivariate calibration and classification problems **[44, 47].**

Kernel PCA is a non-linear variation of PCA that uses the kernel technique as a preprocessing step for classification systems. A non-linear feature selection approach is another name for it. Kernel PCA is an excellent method for detecting nonlinear features in data. Before executing linear PCA on the mapped data, kernel PCA uses a (usually nonlinear) function to convert data into a feature space F **[49, 50].**

**2.3.2 Self-organizing Map (SOM)**

In an n-dimensional array, the SOM, also known as a Kohonen network, translates high-dimensional, non-linear statistical interactions into simple geometric connections **[55]**. Molecular structure descriptors serve as independent variables in a Kohonen self-organizing map, an unsupervised neural network. It can be compared to an elastic net of points adapted to each training compound's unique qualities **[51].** The SOM's neurons compete for selection, which leads to training. These maps were used to visualize the interactions between specific substances and biological receptors **[52].** Because of the system's self-organizing nature, training must be a guided self-learning process. The manifold (Z = [XT YT]) is created during training by attaching the class information of each compound (Y = C1;...; CK) to its descriptor vector (X = x1;...; xD). Only the class index to which the compound belongs is set to 1 in Y, a single-column binary-valued vector holding bioactivity class information. The learned map is then used to categorize novel compounds using this data model, which permits class information to influence the topological ordering of the map during training **[53].**

The topology of the input data and the relative distance between input data are preserved when SOM translates data from a high-dimensional space to a lower-dimensional one. SOM does not require output information during training because it is an unsupervised learning approach; instead, it turns the high-dimensional input space into a two-dimensional map known as the U-Matrix. Nearby neurons are mapped to input data points on the output map next to each other in the input space. As a result, SOM is commonly used to visualize data with many dimensions **[43, 54].**

The Self-Organizing Map (SOM) is a dimensionality reduction technique that allows us to get insights into high-dimensional data quickly. Self-Organizing Maps may be used to analyze exploratory data, solve clustering problems, and visualize large datasets with several dimensions. A pre-defined 2-D lattice of nodes is used to construct SOMs. This node lattice has a structure determined by assigning a location in R^2^ to each node, which is represented as a vector li, the node's index. Each node in the lattice has a position in the input data space, described as a vector wi ∈ Rd, where d is the input data dimension. Each lattice node serves as a link between Rd and R^2^. The structure of the lattice is conserved in a higher-dimensional space **[55,56].**

**2.3.3 Cluster Analysis**

It's yet another tool for breaking down a dataset into subgroups. Cluster analysis is a method of categorizing things into distinct groups or partitioning data into subsets or clusters with comparable qualities shared by the members of the subsets or groups. Data clustering is a common statistical data analysis technique used in various fields, including QSAR research. Hierarchical and partitional data clustering techniques are available. Selecting a distance measure, which determines how the similarity of two elements is calculated, is a critical stage in any grouping. The shape of the clusters will be affected by this **[44, 57].**

**Y scrambling:**

The performance of the original model in data description (r^2^) is compared to that of models created for permuted (randomly shuffled) responses in the validation of QSPR/QSAR models using the y- Randomization tool, depending on the original descriptor pool and the original model construction approach. To avoid the chance of an unintended link, researchers frequently use Y-randomization (also known as y-scrambling or response randomization). It is "possibly the most powerful Validation procedure" **[59].** The significance of the suggested QSAR model is questioned if there is a substantial correlation between selected and randomized response variables. The values below are calculated and recorded during the randomization test: the total number of randomized trials, the non-random trial's R-value, the total number of R values in the random trial that are less than the R-value in the non-random route, the total number of R values from the random path that is higher than the R-value from the non-random track, the confidence level used, R's average value across all random trails, the standard deviation of all random trails' R values from the mean value of R, and the ratio of the mean R-value of all random trials to the non-random R-value in standard deviation. The higher this number, the more probable the nonrandom data model will portray a real link between data variables and behavior **[57].**

The coefficient of determination and RMSE is used to evaluate the QSAR model's effectiveness. Tropsha et al. suggested that the QSAR model should have the following statistical features to be predictive. **[31, 34, 43]:**

q^2^ > 0.5

R^2^ > 0.6

(R^2^ – $R_{0}^{2})$/ R2 < 0.1 or (R2 – $R_{0}^{'2})$/ R^2^ < 0.1

0.85 ≤ *k* ≤ 1.15 or 0.85 ≤ *k’* ≤ 1.15

q^2^ = Cross-validated correlation coefficient

R^2^ = Coefficient of determination (between predicted and observed activities)

$R_{0}^{2}$= The coefficient of determination (predicted versus observed activities)

$R_{0}^{'2}$ = The coefficient of determination (observed versus predicted activities)

k and k’= The slopes of the regression lines through the

Another often-used measure for measuring the relative error of the QSAR model is the root mean squared error (RMSE). The RMS is calculated using the formula below Equation 6 **[34].**
 RMSE = $\sqrt{\sum_{i=1}^{n} \left( x-y \right)^{2}/n}$ …………………. (6)
Where,

RMSE= Root mean square error

X = experimental value of the activity of interest

y = Predicted value of the activity of interest

n = sample size of the data set

Statistical measurements such as the correlation coefficient or the root mean square error (RMSE) can be utilized to evaluate the model's quality. Otherwise, the model will be overfitted if the RMSE is smaller than the RMS of the experimental technique **[33].**

Another form of internal validation is bootstrapping, which involves selecting samples randomly from the data set. Instead of repeatedly investigating data subsets, sub-samples are regularly analyzed in the simplest bootstrapping version. Each sub-sample is a complete sample replacement with a random sample. A typical bootstrap validation selects n items randomly from the original data set to produce K groups of size n. Some of these items may be picked many times in the same random sample, while others may not. The model created from n randomly selected items is used to predict the required attributes for the omitted samples. A high average Q^2^ demonstrates the model's resilience in the bootstrap validation **[60].**

**Pearson coefficient:**

Pearson's correlation coefficient (r) is a widely used statistic for determining the degree to which two variables are connected. r is used to determine the correlation between experimental (x) and predicted (y) values of interest to evaluate the variability that exists between the variables while assessing the relative predictive performance of a QSAR model **[Figure 4]**. By using the following equation (7), the results can be obtained **[34]:**

rxy = $\frac{n\sum xy- \sum x\sum y}{\sqrt{(n\sum x^{2}-({\sum x)}^{2})(n\sum y^{2}-({\sum y)}^{2})}}$ ……………………. (7)

rxy = The correlation coefficient between variables *x* and *y*

*n* = Sample size

*x* = The individual value of variable *x,*

*y* = The individual value of variable *y*

*xy* = The product of variables *x* and *y*

*x^2^* = The squared value of variable *x*

*y^2^* = The squared value of variable *y*

**10-fold cross-validation**:

The method of splitting a data collection into equal parts and utilizing one as the test set and the others as the training set is known as cross-validation (CV) **[Figure 4] [13]**. This approach allows for the sampling and prediction aof ll items in the data set, resulting in an unbiased sample of the data set. Leave-one-out when the number of samples in the data set is restricted; cross-validation is the optimum strategy. The number of folds is the same as the number of samples in the data set. **[ 31, 34, 43].**

Some of the applicability domain methods are explained below.

**Range-based and Geometric Methods**

**Bounding Box-** This approach takes into account the many descriptors that were used to build the model. An n-dimensional hyperrectangle with parallel sides to the coordinate axes is obtained by applying a uniform distribution to each descriptor's greatest and lowest values. This method, however, has numerous drawbacks: because only descriptor ranges are evaluated, unoccupied regions in the interpolation space are not identified, and the connection between descriptors is not taken into account **[65, 66].**

**PCA Bounding Box**- The rotation of axes transforms the initial data into a new orthogonal coordinate system, making compensating for correlations between descriptors easier. PCs with the highest variance in the total dataset are defined as newly formed axes. M-dimensional hyper-rectangle with sides parallel to the PCs defines the spots between each PC's lowest and highest values **[63,65,66].**

**Convex Hull-** This technique employs the smallest convex region encompassing the whole training set to define interpolation space. With increasing data complexity, implementing a Convex Hull can be difficult **[64].** Several solutions are provided for two- or three-dimensional data; nevertheless, the number of dimensions increases the order of complexity. Furthermore, set boundaries are examined without regard for the actual data distribution. Range-based methods, such as Convex Hull, fail to detect potential internal empty spaces within the interpolation space **[65,66].**

**Distance-based Methods-** These methods determine the distance between query chemicals and a certain descriptor space point in the training data. The primary concept is to use a pre-determined threshold to compare distances between a specific location and the dataset. The threshold is a user-defined value that optimizes separating dense areas in the source data. The cut-off number, however, does not accurately reflect the real data density **[64].**

Euclidean and Leverage are two of the most widely used and beneficial distance measurements in QSAR studies. Defining the applicability domain involves calculating the Euclidean distance between all feasible internal and exterior set pairs. When the gap between the new screening compound and its closest neighbor in the internal group is less than the predetermined applicability domain, the prediction of the new screening compound is regarded as appropriate **[31].**

The Euclidean distance is the square root of the squared differences between the corresponding elements of the rows (or columns) in the distance matrix. This is the most prevalent way of distance measurement. The Mahalanobis distance is a weighted Euclidean distance, with the sample variance-covariance matrix determining the weighting. By assuming that the data are evenly distributed, methods based on Euclidean and Mahalanobis distance metrics establish the interpolation zones. Unlike the Euclidean distance, the Mahalanobis distance considers the connection between descriptor axes **[43,65].**

The Mahalanobis distance to the center of the training-set distribution is used in the leverage method. The "hat" matrix is used to compute the leverage h of a chemical reaction, which is Leverage = (xiT (XTX)−1 xi)

Where,

X = The training-set descriptor matrix,

xi = The descriptor vector for the reaction i

The leverage threshold is often calculated as h* = 3*(M + 1)/N, where M is the number of descriptors, and N denotes the number of training samples. Chemical reactions with leverage values h > h* are classified as X-outliers because they are chemically distinct from the training set reactions. Leverage is the name given to this method. The absence of rigorous standards for determining the threshold h* is a disadvantage **[33,63,67].**

**K nearest Neighbors Approach** calculates the degree of similarity between a novel chemical and the compounds in the training space. The distance between a query chemical and the nearest training compound or the distances between k nearest neighbors in the training set is used to estimate similarity **[68,69].**

**Probability Density Distribution-Based Method** is regarded as one of the most sophisticated strategies for determining AD since they are based on computing the Probability Density Function for the input data. These techniques' capacity to recognize internal empty zones is a crucial characteristic. Furthermore, concave zones surrounding the interpolation space borders can be generated if necessary to reflect the real data distribution **[65, 66].** These algorithms generally work by estimating the dataset's probability density first, then finding the Highest Density Region, which is made up of a given proportion (supplied as user input) of the total probability mass **[65].** Each molecule in the training set has a maximum potential that decreases as distance increases. The global potential is generated by adding the individual potentials, representing the probability density **[64]** after calculating all the compounds' potentials.

As shown in **Figure 5**, the AD is significant in all three phases of the (Q)SAR life-cycle The idea should be used during model building to guarantee that a domain is described as broadly as feasible to achieve the required level of predictability. It's worth noting that for a model with a certain number of descriptors, there's usually a trade-off between domain breadth and predictability **[65].**

- - 1. **The methodology of docking**

**2.7.2.1 Flexible docking**

This model keeps the ligand and receptor side chains flexible, and the binding energy for various ligand poses in the receptor is determined. All degrees of freedom in the ligand-receptor combination may theoretically be modeled using MD simulations. However, as previously stated, MD faces the issue of insufficient sampling. The high computational cost of the approach is another barrier, which makes it unsuitable for screening massive chemical databases. Even though It takes a lot of time and costs a lot to compute, it can also predict many possible conformations, making it more versatile. As a result, flexible docking is thought to be a suitable method because it produces better predictions than traditional docking **[70, 75, 86]**

**2.7.2.2 Rigid docking**

Whenever the ligands & receptors both are assumed to be rigid bodies with only three rotational and three translational degrees of freedom, the search space is severely limited. In this case, the flexibility of ligands may be managed by enabling some atom-to-atom overlapping between proteins and the ligands and then using a pre-calculated set of ligand conformation. **[75].** In the method of rigid docking, pharmacophore spheres of different radii are used to depict the ligand and protein binding sites, and also the search algorithms attempt to couple the ligands and protein sphere based on the distance inside the ligand and spheres at protein binding sites **[Figure 9] [86]**. Later, ligands are included in the binding site by fitting the atoms to the sphere centers with the minor square fitting attainable. If the orientation of the ligand isn't proper, it's reoriented till it is. The degree of overlap among the spheres of the ligand and protein pharmacophores is then used for scoring the orientation **[75,86].**

Rigid-body docking simulation has been utilized for virtual screening; this approach is employed as the quickest way to carry out an initial evaluation of a small molecule database. Compared to crystallographic structures, it has a pretty high accuracy **[70,76].**

**Figure 9:** Rigid docking strategy: (A) Protein and ligand. (B) The creation of the initial pose uses a matching point of pharmacophore. The protein and the ligand are supplemented with donor-acceptor and hydrophobic pharmacophore sites. (C) The search algorithm attempts to match the protein and the ligand fitting points by pairing donors with acceptors and hydrophobic atoms with hydrophobic cavities. (D) Various solutions have been discovered.

- - - 1. **Docking of flexible ligands with rigid receptors**

It is essential to consider the flexibility of ligands and receptors as both alter conformations to produce a lower energy's ideal combination in systems that adhere to the given fit paradigm. However, the cost is relatively high when the receptor is also flexible. Docking is usually practiced to treat the ligand as flexible while maintaining the rigidity of the receptor to balance accuracy and computational time. Mostly all docking programs, including AutoDock, have adopted this methodology **[75,86].**

Molecular docking methods are being employed to assess the binding energetics of the predicted ligand-receptor complex. The three categories of scoring functions are force-field-based, experimental, and experience and understanding based**[78].** By combining the contribution of bound ( angle bending, dihedral variation, and bond stretching) and non-bonded (electrostatic and Van der Waals interactions) variables in a generalized master function, force-field-based scoring functions determine the binding energy **[78,87].** Another variation of the evaluation approach uses empirical score functions. The creation of the ligand-receptor complex involves various types of physical processes, each of which is defined by a function word. These comprise ionic and polar interactions, desolvation, entropic effects, hydrogen bonding, and ionic and polar interactions **[88,89].** The knowledge-based scoring functioning is a third way of evaluating ligand-receptor binding energy. The method derives a generic function from paired energy potential derived from an established ligand-receptor complex **[90,91].** These can also represent odd interactions that empirical techniques typically overlook, including sulfur-aromatic or cation **[75].**

Every scoring function has advantages and disadvantages. As a result, the use of multiple scoring systems at the same time has become more common as a means of achieving consensus scores and is highly effective because of the combination of benefits of each strategy while attenuating its drawbacks **[75,78].**

The ligand's structural characteristics, such as translational, rotational, and torsional (dihedral) degrees of freedom, are incrementally adjusted during the conformational search step. This work is carried out using conformational search algorithms, which employ systematically done and stochastic searching approaches **[78, 86].** Systematic search methods encourage minor changes in structure-related characteristics, which progressively change the conformation of the ligands. They are grouped into fragmentation, conformational, and exhaustive ensemble approaches. The critical distinction among them is the ability to deal with the flexibility of ligands **[78,86,92].**

Random search algorithms sample the conformational space by randomly changing a single ligand or a population of ligands. Based on random algorithms, there are three primary types of methods: Genetic Algorithm (GA), Tabu Search (TS), and Monte Carlo (MC) **[86,92].** In molecular docking, numerous search algorithms have been created and are often employed.

**Fast shape matching (SM):**

Shape-matching algorithms consider two molecules overlapping geometrically. Various techniques are used to make multiple arrangements among the ligands and receptors. Because matching algorithms are quick, they can enrich vast libraries with active chemicals. Pharmacophores serve as a representation of the protein and ligand. The distance matrix between the pharmacophore and matching ligand atoms **[75]** determines new ligand conformations; for every matching. It is determined how far the pharmacophore is from the ligand inside the protein. It may be necessary to consider chemical factors, such as hydrogen-bond donors and acceptors, in the calculation. Flexible docking algorithms frequently include the SM approaches as a vital section of the research strategies **[70,75,77].**

**Incremental construction**

This approach divides the ligands into pieces that undergo docking individually in receptor sites. The pieces are fused after the fragments are docked. Because of this fragment forming, the algorithm can be considered ligand-flexible **[Figure 10] [78]**. Rigid docked fragments act as "anchors" that are later joined to flexible ligand portions with rotatable connections. As a result, the receptor binding area, the ligand, is slowly "built." **[75,77,78].**


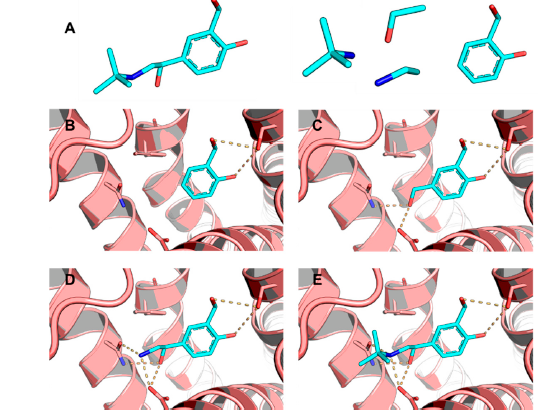


**Figure 10:** The incremental construction method (A) The ligand (stick representation, carbon in cyan) seen to break in numerous fragments; (B) Docking of the anchor fragment in the molecular target's binding site (cartoon representation, carbon in salmon); (C) After the anchor fragment, the subsequent fragment is docked; (D and E) other segments are docked progressively to assemble the full ligand into its binding configuration. A Stick depiction is used to indicate residue in the active site. (carbon in salmon). Hydrogen bonds are depicted as dashed lines.

**Monte Carlo (MC)**

Monte Carlo (MC) procedures form ligand positions by bond rotation or rigid-body translation. The Boltzmann probability function is used in the Monte Carlo stochastic approach to calculate the probability of accepting an unexpected change. Since Monte Carlo (MC) approach uses a simplified energy function and doesn't need derivative information, they have an advantage over molecular dynamics (MD) approaches. **[70,75,78].**

**Genetic algorithm (GA)**

A population of possible solutions is randomly generated via a genetic algorithm process called "chromosome" that records the mapping of ligand fitting sites (such as H-bond atoms) to corresponding protein fitting locations and is encoded for each member of the population. Based on its projected binding affinity, Each chromosome receives a fitness rating, and the population's chromosomes are ordered by fitness score. A point mutation in a chromosome may occur at each step as the operator crossover exchanges data among two of the population chromosomes **[75, 86, 92].**

**Tabu search method**

The binding site is thoroughly searched using the tabu search technique, a Monte Carlo method version that keeps track of the search space of the binding site which has previously been visited **[86].**

1. **Methodologies and Software commonly used in QSAR, pharmacophore modeling, and molecular docking**

**Comparative Molecular Field Analysis (CoMFA):** Camer, in 1988, developed the 3D-QSAR method of Comparative Molecular Field Analysis (CoMFA); it is a significant step forward in the history of QSAR **[Table 4] [21]**. CoMFA illustrates 3D structure-activity connections quantitatively **[18, 21, 101].**

**Comparative Molecular Similarity Indices Analysis (CoMSIA)**

The comparable molecular similarity indices analysis (CoMSIA), a ligand-based sequencing and linear 3D-QSAR technique, is a modified version of CoMFA **[Table 4]**. In CoMSIA, an exponential functional form generated by the SEAL alignment approach replaces the Lennard-Jones and Coulombic potentials used in CoMFA to compute the steric and electrostatic grids. CoMSIA examines a probe's distance-dependent similarity to the atoms that make up the molecule in the database at every grid node. Still, unlike CoMFA, it considers grid nodes inside the molecular volume **[21,102].**

**Molecular Shape Analysis (MSA)**

The method is based on the ligand and 3DQSAR formalism that aims to integrate conformational analysis and the classic Hansch approach **[Table 4]**. The creation of a QSAR model is concerned with the quantitative representation, characterization, and modulation of molecular shape **[21].**

**GRID**

The first program created explicitly for medicinal chemists alternatively, unlike the traditional CoMFA method, was called GRID **[Table 4]**. The GRID fields display how well a target molecule's interaction energy varies with a chemical probe (such as a water probe,  amide probe, or hydrophobic probe) arranged in a 3D grid from around the target. The three interaction forces employed in the GRID force field are hydrogen-bonding acceptor and donor contacts, charged interactions, and induction and dispersion interactions **[18, 21].**

**The Hypothetical Active Site Lattice (HASL)**

This approach uses a grid-based inverse technique to describe the morphologies of the molecule within active places as a group of grid points **[103] [Table 4]**.

**Genetically Evolved Receptor Models (GERM)**

An effective 3D-QSAR approach can be used to produce models that are 3D in nature and that have a macromolecular site to bind without the need for a crystallographically determined or homology-modeled structure of the target receptor **[104] [Table 4].**

**Comparative Binding Energy Analysis (COMBINE)**

It is a technique for computing free binding energy from the 3D structures of complexes of receptors and ligands by deriving a system-specific expression **[Table 4]**. The approach is founded on the idea that binding free energy may link to the grouping of energy-related parts estimated from receptor and ligand structures in bound and unbound states **[21,105].**

**Comparative Molecular Moment Analysis (CoMMA)**

It is among the few alignment-free 3D-QSAR techniques that produce descriptors. Based on charge distributions up to and including second order, related variables, and the geographical moment of molecular mass (shape), the molecular similarity is determined **[106] [Table 4].**

**Comparative Molecular Surface Analysis (CoMSA)**

It's a non-grid 3D-QSAR approach that employs the surface of molecules to determine the areas of the compound compared to mean electrostatic potentials **[21] [Table 4].**

The list of web servers and databases for QSAR drug design research is enlisted in **Table 1 [18,70,100]**. The list of software used for QSAR modeling is enlisted in **Table 2 [19]** and **Table 3 [19]**.

**Molecular Descriptors**

Molecular descriptors are numbers that indicate the features or attributes of a molecule **[47].** The four sorts of descriptions are topological, geometrical, electronic, and hybrid **[19].** The molecular network generates topological descriptors, encapsulating molecular connections into numerical values known as topological indices **[18].** Geometric descriptors encode the molecular structure's three-dimensional characteristics **[54].** Calculating electronic descriptors encodes aspects of the systems associated with the electrons. Charged partial surface area descriptors are a type of hybrid descriptor that encodes a compound's propensity to interact polarly **[19,107] [Table 5 [107], Table 6] [19,100,107].**

1. **Mycobacterium tuberculosis receptors used in all CYP121 approaches**

Of the twenty cytochrome P450 enzymes produced by Mycobacterium tuberculosis, CYP121A1 is the most promising. It is considered worthy of development for antituberculosis medications since it is required for the viability of Mycobacterium tuberculosis H37Rv [114,115].

# DNA gyrase

# Topologically closed DNA molecules can acquire negative supertwists from DNA gyrases. DNA gyrase and Topo IV (topoisomerase IV) are type IIA topoisomerases found in most bacteria, including E. coli and B. subtilis, which serve as model organisms. Mycobacterium tuberculosis, the human pathogen that causes tuberculosis, has only one type IIA topoisomerase, DNA gyrase, according to the first full genome sequencing [116,117,118].

# Mycobacterium membrane protein Large 3 (MmpL3)

# It is a trehalose monomycolate cell membrane transporter, which is an introduction to the manufacturing of mycolic acid, which is required for the creation of the cell wall of bacteria and is harmful [119,120].

# DprE1

# DprE (decaprenylphosphoryl-D-ribose 20-epimerase) is necessary for developing and maintaining the TB cell walls of Mycobacteria. DprE1 is an arabinose sugar donate that helps the Decaprenyl-phosphoryl D-arabinose (DPA) pathway produce cell wall components like lipoarabinomannan and arabinogalactan [121].

# Mannose receptor

# The macrophage mannose receptor is a transmembrane protein that is monomeric. Mannose receptors on human monocyte-derived macrophages allow them to adhere to and absorb pathogenic Mycobacterium TB. The dangerous strains Erdman and H37Rv bind mannose receptors, not the avirulent H37Ra strain.

# Sp-A and Sp-A receptors

# M. tuberculosis binding and absorption are improved by surfactant protein A (Sp-A). Purified Sp-A binds directly to M. tuberculosis H37Ra, with calcium and glycosylation of Sp-A required for bacterial binding [122].

# Mycobacterium TB phagocytosis utilizes a variety of receptors, including scavenger receptors, mannose receptors (MR), and complement receptors. Pattern recognition receptors (PRRs) recognize distinctive pathogen-associated molecular patterns (PAMPs) of mycobacteria and generate intracellular signals that lead to the generation of cytokines and the commencement of adaptive immunity [123]. PRRs detect the presence of bacteria, fungi, and viruses. These families involve transmembrane proteins like TLRs and c-type lectin receptors (CTLRs), as well as RIG-I-like receptors (RLRs) and NOD-like receptors are cytoplasmic proteins (NLRs) [124].

# Toll-like receptors

# Mycobacterium tuberculosis interacts with TLRs to activate phagocytes; however, this does not lead to fast mycobacterial engulfment. Signaling pathways are initiated when specific mycobacterial structures link with TLRs, and Myeloid differentiating major response protein 88 (MyD88), an adapter molecule, is essential. MyD88 is required for Mycobacterium tuberculosis innate immune response activation [125]. TLR2, TLR4, TLR9, and potentially TLR8 are known to be involved in the identification of MTB [126].

# Nod (nucleotide oligomerization domain)-like receptor,- The NOD-like receptors (NLRs) protein family is quite similar to the plant R (resistance) protein family, which has a vital role in plant pathogen defense. MTB increases proinflammatory cytokine production via NOD2, an intracellular receptor. NOD2 is a peptidoglycan receptor found in bacteria [123,127].

# Scavenger receptors

Polyanionic macromolecules and particles, such as lipoteichoic acid and lipopolysaccharides of gram-positive and gram-negative bacteria, bind to macrophage scavenger receptors. Mammalian macrophages and monocytes include scavenger receptors (SRs), which may recognize a wide range of ligands **[123,128,129].**

1. Yuanita, E., Sudirman, Dharmayani, N. K. T., Ulfa, M., & Syahri, J. (2020). Quantitative structure-activity relationship (QSAR) and molecular docking of xanthone derivatives as anti-tuberculosis agents. Journal of Clinical Tuberculosis and Other Mycobacterial Diseases, 21, 100203. <https://doi.org/10.1016/j.jctube.2020.100203>.
2. Vastrad, C. M. (2012). Predictive comparative QSAR analysis of sulfathiazole analogs as Mycobacterium tuberculosis H37Rv. Health Informatics- An International Journal (HIIJ), 3, 379-390. <https://doi.org/10.48550/arXiv.1402.5466>.
3. Adeniji, S. E., Uba, S., & Uzairu, A. (2018). QSAR modeling and molecular docking analysis of some active compounds against Mycobacterium tuberculosis receptor (Mtb CYP121). Journal of Pathogens, 2018, 1-24. https://doi.org/10.1155/2018/1018694.
4. Dwivedi, N., Mishra, B. N., & Katoch, V. M. (2011). 2D-QSAR model development and analysis on variant groups of anti-tuberculosis drugs. Bioinformation, 7, 82-90. doi:10.6026/97320630007082.
5. Shetye, G. S., Franzblau, S. G., & Cho, S. (2020). New tuberculosis drug targets, their inhibitors, and potential therapeutic impact. Translational Research, 220, 68-97. doi:10.1016/j.trsl.2020.03.007.
6. Chapman, T. M., Bouloc, N., Buxton, R. S., Chugh, J., Lougheed, K. E., Osborne, S. A., Saxty, B., Smerdon, S. J., Taylor, D. L., Whalley, D. (2012). Substituted aminopyrimidine protein kinase B (PknB) inhibitors show activity against Mycobacterium tuberculosis. Bioorganic & Medicinal Chemistry Letters, 22(9), 3349-3353. https://doi.org/10.1016/j.bmcl.2012.02.107.
7. Ahamad, S., Rahman, S., Khan, F. I., Dwivedi, N., Ali, S., Kim, J., & Hassan, M. I. (2017). QSAR-based therapeutic management of M. tuberculosis. Archives of Pharmacal Research, 40, 676-694. doi:10.1007/s12272-017-0914-1.
8. Martins, F., Ventura, C., Santos, S., & Viveiros, M. (2013). QSAR-based design of new antitubercular compounds: improved isoniazid derivatives against multidrug-resistant TB. Current Pharmaceutical Design, 20, 4427-4454. doi:10.2174/1381612819666131118164434.
9. Jin, Y., Fan, S., Lv, G., Meng, H., Sun, Z., Jiang, W., Van Lanen, S. G., & Yang, Z. (2017). Computer-aided drug design of rapamycin analogues as anti-tuberculosis antibiotics by 3D-QSAR and molecular docking. Open Chemistry, 15, 299-307. doi:10.1515/chem-2017-0039.
10. Doreswamy, H., & Vastrad, C. M. (2013). Predictive comparative QSAR analysis of 5-nitrofuran-2-yl derivatives Myco Bacterium tuberculosis H37RV inhibitors. Health Informatics - An International Journal, 2, 47-62. doi:10.5121/hiij.2013.2404.
11. Ojo, O. A., Ojo, A. B., Okolie, C., Nwakama, M. A. C., Iyobhebhe, M., Evbuomwan, I. O., Nwonuma, C. O., Maimako, R. F., Adegboyega, A. E., Taiwo, O. A., Alsharif, K. F., & Batiha, G. E. S. (2021). Deciphering the interactions of bioactive compounds in selected traditional medicinal plants against Alzheimer's diseases via pharmacophore modeling, auto-QSAR, and molecular docking approaches. Molecules, 26. doi:10.3390/molecules26071996.
12. Adeniji, S. E., Uba, S., & Uzairu, A. (2018). In silico study for investigating and predicting the activities of 1,2,4-triazole derivatives as potent anti-tubercular agents. The Journal of Engineering and Exact Sciences, 4, 0246-0254. doi:10.18540/jcecvl4iss2pp0246-0254.
13. Cronin, M. T. D. D., Jaworska, J. S., Walker, J. D., Comber, M. H. I., Watts, C. D., Worth, A. P. P., ... Mohimont, L. (2008). Report of the EPAA-ECVAM workshop on the validation of Integrated Testing Strategies (ITS). Alternatives to Laboratory Animals: ATLA, 27, 258-284.
14. Qing, X., Lee, X. Y., De Raeymaeker, J., Tame, J. R., Zhang, K. Y., De Maeyer, M., & Voet, A. R. (2014). Pharmacophore modeling: Advances, limitations, and current utility in drug discovery. Journal of Receptor, Ligand, and Channel Research, 7, 81-92. https://doi.org/10.2147/JRLCR.S46843.
15. Macalino, S. J. Y., Billones, J. B., Organo, V. G., & Carrillo, M. C. O. (2020). In silico strategies in tuberculosis drug discovery. Molecules, 25(3), 665. https://doi.org/10.3390/molecules25030665.
16. Leach, A. R., Gillet, V. J., Lewis, R. A., & Taylor, R. (2010). Three-dimensional pharmacophore methods in drug discovery. Journal of Medicinal Chemistry, 53, 539-558. https://doi.org/10.1021/jm900817u.
17. Wermuth, C. G., Ganellin, C. R., Lindberg, P., & Mitscher, L. A. (1998). Glossary for chemists of terms used in medicinal chemistry. Pure & Applied Chemistry, 70, 1129-1143.
18. Bajot, F. (2010). The use of QSAR and computational methods in drug design. Challenges and Advances in Computational Chemistry and Physics, 8, 261-282. DOI: 10.1007/978-1-4020-9783-6_9.
19. Abdel-Ilah, L., Veljović, E., Gurbeta, L., & Badnjević, A. (2017). Applications of QSAR study in drug design. 6, 582-587.
20. Kwon, S., Bae, H., Jo, J., & Yoon, S. (2019). Comprehensive ensemble in QSAR prediction for drug discovery. BMC Bioinformatics, 20(1), 521. https://doi.org/10.1186/s12859-019-3135-4.
21. Verma, J., Khedkar, V., & Coutinho, E. (2010). 3D-QSAR in drug design - A review. Current Topics in Medicinal Chemistry, 10, 95-115. https://doi.org/10.2174/156802610790232260.
22. Testa, B. (1995). QSAR: Hansch analysis and related approaches. Trends in Pharmacological Sciences, 16, 280.
23. Tandon, H., Chakraborty, T., & Suhag, V. (2019). A concise review on the significance of QSAR in drug design. Chemical and Biomolecular Engineering, 4, 45.
24. Neves, B. J., Braga, R. C., Melo-Filho, C. C., Moreira-Filho, J. T., Muratov, E. N., & Andrade, C. H. (2018). QSAR-based virtual screening: Advances and applications in drug discovery. Frontiers in Pharmacology. https://doi.org/10.3389/fphar.2018.01275.
25. Cherkasov, A., Muratov, E. N., Fourches, D., Varnek, A., Baskin, I. I., Cronin, M. T., ... Tropsha, A. (2014). QSAR modeling: Where have you been? Where are you going to? Journal of Medicinal Chemistry, 57(12), 4977-5010. https://doi.org/10.1021/jm4004285.
26. Winkler, D. A. (2002). The role of quantitative structure-activity relationships (QSAR) in biomolecular discovery. Briefings in Bioinformatics, 3, 73-86. https://doi.org/10.1093/bib/3.1.73.
27. Aparoy, P., Reddy, K. K., & Reddanna, P. (2012). Structure and ligand based drug design strategies in the development of novel 5-LOX inhibitors. Current Medicinal Chemistry, 19, 3763-3778. https://doi.org/10.2174/092986712801661112.
28. Gandhi, A., Masand, V., Zaki, M. E. A., Al-Hussain, S. A., Ghorbal, A. B., & Chapolikar, A. (2021). Quantitative structure-activity relationship evaluation of MDA-MB-231 cell anti-proliferative leads. Molecules, 26(16), 4795. https://doi.org/10.3390/molecules26164795.
29. Munteanu, C. R., Fernandez-Blanco, E., Seoane, J. A., Izquierdo-Novo, P., Rodriguez-Fernandez, J. A., Prieto-Gonzalez, J. M., ... Pazos, A. (2010). Drug discovery and design for complex diseases through QSAR computational methods. Current Pharmaceutical Design, 16, 2640-2655. https://doi.org/10.2174/138161210792389252.
30. Patel, H. M., Noolvi, M. N., Sharma, P., Jaiswal, V., Bansal, S., Lohan, S., ... Bhardwaj, V. (2014). Quantitative structure-activity relationship (QSAR) studies as a strategic approach in drug discovery. Medicinal Chemistry Research, 23, 4991-5007. DOI: 10.2174/1573406415666190513100646.
31. Prachayasittikul, V., Worachartcheewan, A., Shoombuatong, W., Songtawee, N., Simeon, S., Prachayasittikul, V., & Nantasenamat, C. (2015). Computer-aided drug design of bioactive natural products. Current Topics in Medicinal Chemistry, 15, 1780-1800. DOI: 10.2174/1568026615666150506151101.
32. Piir, G., Kahn, I., García-Sosa, A. T., Sild, S., Ahte, P., & Maran, U. (2018). Best practices for QSAR model reporting: Physical and chemical properties, ecotoxicity, environmental fate, human health, and toxicokinetics endpoints. Environmental Health Perspectives, 126, 1-20. https://doi.org/10.1289/EHP3264.
33. Schaduangrat, N., Lampa, S., Simeon, S., Gleeson, M. P., Spjuth, O., & Nantasenamat, C. (2020). Towards reproducible computational drug discovery. Journal of Cheminformatics, 12, 1-30. https://doi.org/10.1186/s13321-020-0408-x.
34. Nantasenamat, C., Isarankura-Na-Ayudhya, C., Naenna, T., & Prachayasittikul, V. (2009). A practical overview of quantitative structure-activity relationship. EXCLI Journal, 8, 74-88.
35. Naveja, J. J., & Medina-Franco, J. L. (2019). Finding constellations in chemical space through core analysis. Frontiers in Chemistry, 7, 1-10. https://doi.org/10.3389/fchem.2019.00510.
36. Ganesan, A. (2008). The impact of natural products upon modern drug discovery. Current Opinion in Chemical Biology, 12, 306-317. https://doi.org/10.1016/j.cbpa.2008.03.016.
37. Benet, L. Z., Hosey, C. M., Ursu, O., & Oprea, T. I. (2016). BDDCS, the Rule of 5, and drugability. Advanced Drug Delivery Reviews, 101, 89-98. https://doi.org/10.1016/j.addr.2016.05.007.
38. Lambrinidis, G., & Tsantili-Kakoulidou, A. (2018). Challenges with multi-objective QSAR in drug discovery. Expert Opinion on Drug Discovery, 13, 851-859. https://doi.org/10.1080/17460441.2018.1496079.
39. Koch, M. A., Schuffenhauer, A., Scheck, M., Wetzel, S., Casaulta, M., Odermatt, A., ... Weldmann, H. (2005). Charting biologically relevant chemical space: A structural classification of natural products (SCONP). Proceedings of the National Academy of Sciences of the United States of America, 102, 17272-17277. https://doi.org/10.1073/pnas.0503647102.
40. Reayi, A., & Arya, P. (2005). Natural product-like chemical space: Search for chemical dissectors of macromolecular interactions. Current Opinion in Chemical Biology, 9, 240-247. https://doi.org/10.1016/j.cbpa.2005.04.007
41. Rosén, J., Gottfries, J., Muresan, S., Backlund, A., & Oprea, T. I. (2009). Novel chemical space exploration via natural products. Journal of Medicinal Chemistry, 52, 1953-1962. https://doi.org/10.1021/jm801514w.
42. Zhou, X., Li, Y., & Chen, X. (2010). Computational identification of bioactive natural products by structure-activity relationship. Journal of Molecular Graphics and Modelling, 29, 38-45. https://doi.org/10.1016/j.jmgm.2010.04.007.
43. Nantasenamat, C., Isarankura-Na-Ayudhya, C., & Prachayasittikul, V. (2010). Advances in computational methods to predict the biological activity of compounds. Expert Opinion on Drug Discovery, 5, 633-654. https://doi.org/10.1517/17460441.2010.492827.
44. Pirhadi, S., Shiri, F., & Ghasemi, J. B. (2015). Multivariate statistical analysis methods in QSAR. RSC Advances, 5, 104635-104665. https://doi.org/10.1039/C5RA10729F.
45. Jollife, I. T., & Cadima, J. (2016). Principal component analysis: A review and recent developments. Philosophical Transactions of the Royal Society A: Mathematical, Physical and Engineering Sciences, 374. https://doi.org/10.1098/rsta.2015.0202.
46. Estimation, E. E., Logistic, F., Author, R., Source, G. G., & Bootstrap: Forward Error Regression. Journal of the American Statistical Association, 81, 108-113.
47. Saha, P., Roy, N., Mukherjee, D., & Sarkar, A. K. (2016). Application of Principal Component Analysis for Outlier Detection in Heterogeneous Traffic Data. Procedia Computer Science, 83, 107-114. https://doi.org/10.1016/j.procs.2016.04.105.
48. Chen, X., Zhang, B., Wang, T., Wang, T., Bonni, A., & Zhao, G. (2020). Robust principal component analysis for accurate outlier sample detection in RNA-Seq data. BMC Bioinformatics, 21, 1-20. https://doi.org/10.1186/s12859-020-03608-0.
49. Begam, B. F., & Rajeswari, J. (2019). Visualization of chemical space using Kernel-based principal component research. International Journal of Innovative Technology and Exploring Engineering, 8, 590-593. DOI: 10.35940/ijitee.K1097.09811S19.
50. Alsenan, S. A., Al-Turaiki, I. M., & Hafez, A. M. (2020). Feature extraction methods in quantitative structure-activity relationship modeling: A comparative study. IEEE Access, 8, 78737-78752. DOI: 10.1109/ACCESS.2020.2990375.
51. Guha, R., Serra, J. R., & Jurs, P. C. (2004). Generation of QSAR sets with a self-organizing map. Journal of Molecular Graphics and Modelling, 23, 1-14. https://doi.org/10.1016/j.jmgm.2004.03.003.
52. Polański, J. (2000). Self-organizing neural network for modeling 3D QSAR of colchicinoids. Acta Biochimica Polonica, 47, 37-45.
53. Bayram, E., Santago, P., Harris, R., Xiao, Y. D., Clauset, A. J., & Schmitt, J. D. (2004). Genetic algorithms and self-organizing maps: A powerful combination for modeling complex QSAR and QSPR problems. Journal of Computer-Aided Molecular Design, 18, 483-493. https://doi.org/10.1007/s10822-004-5321-2.
54. Paper, I., Introduction, N., Models, S.-o., Maps, K., & Kohen self-organization map. Proceedings of the IEEE, 78, 1464-1480.
55. Ponmalai, R., & Kamath, C. (2019). Self-Organizing Maps and Their Applications to Data Analysis. Lawrence Livermore National Laboratory, 46. https://doi.org/10.2172/1566795.
56. Fiannaca, A., Di Fatta, G., Rizzo, R., Urso, A., & Gaglio, S. (2007). Fast training of self-organizing maps for the visual exploration of molecular compounds. IEEE International Conference on Neural Networks - Conference Proceedings, 2776-2781. http://dx.doi.org/10.1109/IJCNN.2007.4371399.
57. Mahobia, N. K., Patel, R. D., Sheikh, N. W., Singh, S. K., Mishra, A., & Dhardubey, R. (2010). Validation Method Used In Quantitative Structure Activity Relationship. Der Pharma Chemica, 2, 260-271.
58. Geng, H., Chen, F., Ye, J., & Jiang, F. (2019). Applications of Molecular Dynamics Simulation in Structure Prediction of Peptides and Proteins. Computational and Structural Biotechnology Journal, 17, 1162-1170. https://doi.org/10.1016/j.csbj.2019.07.010.
59. Rücker, C., Rücker, G., & Meringer, M. (2007). Y-randomization and its variants in QSPR/QSAR. Journal of Chemical Information and Modeling, 47, 2345-2357. https://doi.org/10.1021/ci700157b.
60. Veerasamy, R., Rajak, H., Jain, A., Sivadasan, S., Varghese, C. P., & Agrawal, R. K. (2011). Validation of QSAR Models - Strategies and Importance. International Journal of Drug Design and Discovery, 2, 511-519.
61. Weaver, S., & Gleeson, M. P. (2008). The importance of the domain of applicability in QSAR modeling. Journal of Molecular Graphics and Modelling, 26, 1315-1326. https://doi.org/10.1016/j.jmgm.2008.01.002.
62. Sushko, I. (2011). Applicability domain of QSAR models.
63. Varsou, D. D., Nikolakopoulos, S., Tsoumanis, A., Melagraki, G., & Afantitis, A. (2018). Discovery and computational toxicology. In Computational Toxicology (pp. 287-311). Springer. DOI: 10.1007/978-1-4939-7899-1_14.
64. Sahigara, F., Mansouri, K., Ballabio, D., Mauri, A., Consonni, V., & Todeschini, R. (2012). Comparison of different approaches to define the applicability domain of QSAR models. Molecules, 17, 4791-4810. https://doi.org/10.3390/molecules17054791.
65. Netzeva, T. I., Worth, A. P., Aldenberg, T., et al. (2005). Current status of methods for defining the applicability domain of (quantitative) structure-activity relationships. ATLA Alternatives to Laboratory Animals, 33, 155-173. https://doi.org/10.1177/026119290503300209.
66. Jaworska, J., Nikolova-Jeliazkova, N., & Aldenberg, T. (2005). QSAR applicability domain estimation by projection of the training set in descriptor space: A review. ATLA Alternatives to Laboratory Animals, 33, 445-459. https://doi.org/10.1177/0261192905033005.
67. Rakhimbekova, A., Madzhidov, T. I., Nugmanov, R. I., Gimadiev, T. R., Baskin, I. I., & Varnek, A. (2020). Comprehensive analysis of applicability domains of QSPR models for chemical reactions. International Journal of Molecular Sciences, 21, 1-20. https://doi.org/10.3390/ijms21155542.
68. Sheridan, R. P., Feuston, B. P., Maiorov, V. N., & Kearsley, S. K. (2004). Similarity to molecules in the training set is a good discriminator for prediction accuracy in QSAR. Journal of Chemical Information and Computer Sciences, 44, 1912-1928. https://doi.org/10.1021/ci049782w.
69. Mathea, M., Klingspohn, W., & Baumann, K. (2016). Chemoinformatic Classification Methods and their Applicability Domain. Molecular Informatics, 35, 160-180. https://doi.org/10.1002/minf.201501019.
70. Kumar, A., & S, P. (2018). A Review on Rational Drug Design Approach: Novel Tool for Drug Discovery. International Journal of Research in Ayurveda and Pharmacy, 9, 17-21.
71. Yang, S. Y. (2010). Pharmacophore modeling and applications in drug discovery: Challenges and recent advances. Drug Discovery Today, 15, 444-450. https://doi.org/10.1016/j.drudis.2010.03.013.
72. Muhammed, M. T., & Aki-Yalcin, E. (2021). Pharmacophore modeling in drug discovery: Methodology and current status. Journal of the Turkish Chemical Society, Section A: Chemistry, 8, 749-762. https://doi.org/10.18596/jotcsa.927426.
73. Mesli, F., Ghalem, M., Daoud, I., & Ghalem, S. (2021). Potential inhibitors of angiotensin-converting enzyme 2 receptor of COVID-19 by Corchorus olitorius Linn using docking, molecular dynamics, conceptual DFT investigation, and pharmacophore mapping. Journal of Biomolecular Structure and Dynamics, 0, 1-13. https://doi.org/10.1080/07391102.2021.1896389.
74. Seidel, T., Wieder, O., Garon, A., & Langer, T. (2020). Applications of the Pharmacophore Concept in Natural Product-inspired Drug Design. Molecular Informatics. https://doi.org/10.1002/minf.202000059.
75. Meng, X. Y., Zhang, H. X., Mezei, M., & Cui, M. (2012). Molecular Docking: A Powerful Approach for Structure-Based Drug Discovery. Current Computer Aided-Drug Design, 7, 146-157. DOI: 10.2174/157340911795677602.
76. Dar, A. M., & Mir, S. (2017). Molecular Docking: Approaches, Types, Applications, and Basic Challenges. Journal of Analytical & Bioanalytical Techniques, 8, 10. DOI: 10.4172/2155-9872.1000356.
77. Wang, G., & Zhu, W. (2016). Molecular docking for drug discovery and development: A widely used approach but far from perfect. Future Medicinal Chemistry. https://doi.org/10.4155/fmc-2016-0143.
78. Ferreira, L. G., Dos Santos, R. N., Oliva, G., & Andricopulo, A. D. (2015). Molecular docking and structure-based drug design strategies. Molecules, 20, 13384-13421. https://doi.org/10.3390/molecules200713384.
79. Kumalo, H. M., Bhakat, S., & Soliman, M. E. S. (2015). Theory and applications of covalent docking in drug discovery: Merits and pitfalls. Molecules, 20, 1984-2000. https://doi.org/10.3390/molecules20021984.
80. Singh, J., Petter, R. C., Baillie, T. A., & Whitty, A. (2011). The resurgence of covalent drugs. Nature Reviews Drug Discovery, 10, 307-317. https://doi.org/10.1038/nrd3410.
81. Hartshorn, M. J., Verdonk, M. L., Chessari, G., Brewerton, S. C., Mooij, W. T. M., Mortenson, P. N., & Murray, C. W. (2007). Diverse, high-quality test set for the validation of protein-ligand docking performance. Journal of Medicinal Chemistry, 50, 726-741. https://doi.org/10.1021/jm061277y.
82. Katritch, V., Byrd, C. M., Tseitin, V., Dai, D., Raush, E., Totrov, M., Abagyan, R., Jordan, R., & Hruby, D. E. (2007). Discovery of small molecule inhibitors of ubiquitin-like poxvirus proteinase I7L using homology modeling and covalent docking approaches. Journal of Computer-Aided Molecular Design, 21, 549-558. https://doi.org/10.1007/s10822-007-9138-7.
83. Wang, Q., Mach, R. H., & Reichert, D. E. (2009). Docking and 3D-QSAR studies on isatin sulfonamide analogues as caspase-3 inhibitors. Journal of Chemical Information and Modeling, 49, 1963-1973. https://doi.org/10.1021/ci900144x.
84. Ma, Y., Xu, B., Fang, Y., Yang, Z., Cui, J., Zhang, L., & Zhang, L. (2011). Synthesis and SAR study of novel peptide aldehydes as inhibitors of 20S proteasome. Molecules, 16, 7551-7564. https://doi.org/10.3390/molecules16097551.
85. Lawandi, J., Toumieux, S., Seyer, V., Campbell, P., Thielges, S., Juillerat-Jeanneret, L., & Moitessier, N. (2009). Constrained peptidomimetics reveal detailed geometric requirements of covalent prolyl oligopeptidase inhibitors. Journal of Medicinal Chemistry, 52, 6672-6684. https://doi.org/10.1021/jm901013a.
86. Stanzione, F., Giangreco, I., & Cole, J. C. (2021). Use of molecular docking computational tools in drug discovery. Progress in Medicinal Chemistry, 60, 273-343. https://doi.org/10.1016/bs.pmch.2021.01.004.
87. Englebienne, P., & Moitessier, N. (2009). Docking ligands into flexible and solvated macromolecules. 5. Force-field-based prediction of binding affinities of ligands to proteins. Journal of Chemical Information and Modeling, 49, 2564-2571. https://doi.org/10.1021/ci900251k.
88. Murray, C. W., Auton, T. R., & Eldridge, M. D. (1998). Empirical scoring functions. II. The testing of an empirical scoring function for the prediction of ligand-receptor binding affinities and the use of Bayesian regression to improve the quality of the model. Journal of Computer-Aided Molecular Design, 12, 503-519. https://doi.org/10.1023/A:1008040323669.
89. Eldridge, M. D., Murray, C. W., Auton, T. R., Paolini, G. V., & Mee, R. P. (1997). Empirical scoring functions: I. The development of a fast empirical scoring function to estimate the binding affinity of ligands in receptor complexes. Journal of Computer-Aided Molecular Design, 11, 425-445. https://doi.org/10.1023/A:1007996124545.
90. Allouche, A. R. (2012). Software News and Updates Gabedit — A Graphical User Interface for Computational Chemistry Software. Journal of computational chemistry, 32, 174-182. https://doi.org/10.1002/jcc.21600.
91. Gohlke, H., Hendlich, M., & Klebe, G. (2000). Knowledge-based scoring function to predict protein-ligand interactions. Journal of Molecular Biology, 295, 337-356. https://doi.org/10.1006/jmbi.1999.3371.
92. Sanchez, G. (2013). Las instituciones de ciencia y tecnología en los procesos de aprendizaje de la producción agroalimentaria en Argentina. El sistema argentino de innovación: instituciones, empresas y redes. El desafío de la creación y apropiación de conocimiento, 14, 659-664.
93. Huang, H. J., Yu, H. W., Chen, C. Y., Hsu, C. H., Chen, H. Y., Lee, K. J., Tsai, F. J., & Chen, C. Y. C. (2010). Current developments of computer-aided drug design. Journal of the Taiwan Institute of Chemical Engineers, 41, 623-635. DOI: 10.1016/j.jtice.2010.03.017.
94. Khan, F. I., Wei, D. Q., Gu, K. R., Hassan, M. I., & Tabrez, S. (2016). Current updates on computer-aided protein modeling and designing. International Journal of Biological Macromolecules, 85, 48-62. https://doi.org/10.1016/j.ijbiomac.2015.12.072.
95. De Vivo, M., Masetti, M., Bottegoni, G., & Cavalli, A. (2016). Role of Molecular Dynamics and Related Methods in Drug Discovery. Journal of Medicinal Chemistry, 59, 4035-4061. https://doi.org/10.1021/acs.jmedchem.5b01684.
96. Harvey, M. J., & De Fabritiis, G. (2012). High-throughput molecular dynamics: The powerful new tool for drug discovery. Drug Discovery Today, 17, 1059-1062. https://doi.org/10.1016/j.drudis.2012.03.017.
97. Good, A. (2006). Virtual screening. Comprehensive Medicinal Chemistry II, 4, 459-494.
98. Hospital, A., Goñi, J. R., Orozco, M., & Gelpí, J. L. (2015). Molecular dynamics simulations: Advances and applications. Advances and Applications in Bioinformatics and Chemistry, 8, 37-47. https://doi.org/10.2147/AABC.S70333.
99. Hansson, T., Oostenbrink, C., & Van Gunsteren, W. F. (2002). Molecular dynamics simulations. Current Opinion in Structural Biology, 12, 190-196. https://doi.org/10.1016/S0959-440X(02)00308-1.
100. Singla, D., Dhanda, S. K., Chauhan, J. S., Bhardwaj, A., Brahmachari, S. K., & Raghava, G. P. S. (2013). Open Source Software and Web Services for Designing Therapeutic Molecules. Current Topics in Medicinal Chemistry, 13, 1172-1191. https://doi.org/10.2174/1568026611313100005.
101. Yang, L. B., Jiang, D. Q., Qi, W. B., Zhang, T., Feng, Y. L., Gao, L., & Zhao, J. (2012). Subclinical hyperthyroidism and the risk of cardiovascular events and all-cause mortality: An updated meta-analysis of cohort studies. European Journal of Endocrinology, 167, 75-84. <https://doi.org/10.1530/EJE-12-0015>.
102. Doweyko, A. M. (2006). Three-Dimensional quantitative structure-activity relationship: The state of the art. Comprehensive Medicinal Chemistry II, 4, 575-595.
103. Doweyko, A. M. (1988). The Hypothetical Active Site Lattice. An Approach to Modelling Active Sites from Data on Inhibitor Molecules. Journal of Medicinal Chemistry, 31, 1396-1406. https://doi.org/10.1021/jm00402a025.
104. Walters, D. E., Hinds, R. M. (1994). Genetically Evolved Receptor Models: A Computational Approach to Construction of Receptor Models. Journal of Medicinal Chemistry, 37, 2527-2536. https://doi.org/10.1021/jm00042a006.
105. Henrich, S., Feierberg, I., Wang, T., Blomberg, N., Wade, R. C. (2010). Comparative binding energy analysis for binding affinity and target selectivity prediction. Proteins: Structure, Function and Bioinformatics, 78, 135-153. https://doi.org/10.1002/prot.22579.
106. Silverman, B. D., Platt, D. E. (1996). Comparative molecular moment analysis (coMMA): 3D-QSAR without molecular superposition. Journal of Medicinal Chemistry, 39, 2129-2140. https://doi.org/10.1021/jm950589q.
107. Danishuddin, Khan, A. U. (2016). Descriptors and their selection methods in QSAR analysis: paradigm for drug design. Drug Discovery Today, 21, 1291-1302. https://doi.org/10.1016/j.drudis.2016.06.013.
108. Baroni, M., Cruciani, G., Sciabola, S., Perruccio, F., Mason, J. S. (2007). A common reference framework for analyzing/comparing proteins and ligands. Fingerprints for Ligands and Proteins (FLAP): Theory and application. Journal of Chemical Information and Modeling, 47, 279-294. https://doi.org/10.1021/ci600253e.
109. Schaller, D., Šribar, D., Noonan, T., Deng, L., Nguyen, T. N., Pach, S., Machalz, D., Bermudez, M., Wolber, G. (2020). Next generation 3D pharmacophore modeling. Wiley Interdisciplinary Reviews: Computational Molecular Science, 10, 1-20. https://doi.org/10.1002/wcms.1468.
110. Pagadala, N. S., Syed, K., Tuszynski, J. (2017). Software for molecular docking: a review. Biophysical Reviews, 9, 91-102. https://doi.org/10.1007/s12551-016-0247-1.
111. Zhang, B., Li, H., Yu, K., Jin, Z. (2022). Molecular docking-based computational platform for high-throughput virtual screening. CCF Transactions on High Performance Computing, 4, 63-74. https://doi.org/10.1007/s42514-021-00086-5.
112. Scarpino, A., Ferenczy, G. G., Keserü, G. M. (2018). Comparative Evaluation of Covalent Docking Tools. Journal of Chemical Information and Modeling, 58, 1441-1458. https://doi.org/10.1021/acs.jcim.8b00228.
113. Myint, K. Z., Wang, L., Tong, Q., Xie, X. Q. (2012). Molecular fingerprint-based artificial neural networks QSAR for ligand biological activity predictions. Molecular Pharmaceutics, 9, 2912-2923. https://doi.org/10.1021/mp300237z.
114. Ortiz de Montellano, P. R. (2018). Potential drug targets in the Mycobacterium tuberculosis cytochrome P450 system. Journal of Inorganic Biochemistry, 180, 235-245. https://doi.org/10.1016/j.jinorgbio.2018.01.010.
115. Hudson, S. A., McLean, K. J., Surade, S., Yang, Y. Q., Leys, D., Ciulli, A., Munro, A. W., Abell, C. (2012). Application of fragment screening and merging to the discovery of inhibitors of the Mycobacterium tuberculosis cytochrome P450 CYP121. Angewandte Chemie - International Edition, 51, 9311-9316. https://doi.org/10.1002/anie.201202544.
116. Bouige, A., Darmon, A., Piton, J., Roue, M., Petrella, S., Capton, E., Forterre, P., Aubry, A., Mayer, C. (2013). Mycobacterium tuberculosis DNA gyrase possesses two functional GyrA-boxes. Biochemical Journal, 455, 285-294. https://doi.org/10.1042/BJ20130430.
117. Nagaraja, V., Godbole, A. A., Henderson, S. R., Maxwell, A. (2017). DNA topoisomerase I and DNA gyrase as targets for TB therapy. Drug Discovery Today, 22, 510-518. https://doi.org/10.1016/j.drudis.2016.11.006.
118. Mdluli, K., Ma, Z. (2008). Mycobacterium tuberculosis DNA Gyrase as a Target for Drug Discovery. Infectious Disorders - Drug Targets, 7, 159-168. https://doi.org/10.2174/187152607781001763.
119. Mdluli, K., Kaneko, T., Upton, A. (2015). The Tuberculosis Drug Discovery and Development Pipeline and Emerging drug targets. Drug Cold Spring Harb. Perspect. Med., 5(6), a021154. https://doi.org/10.1101/cshperspect.a021154.
120. Umare, M. D., Khedekar, P. B., Chikhale, R. V. (2021). Mycobacterial Membrane Protein Large 3 (MmpL3) Inhibitors: A Promising Approach to Combat Tuberculosis. ChemMedChem, 16, 3136-3148. https://doi.org/10.1002/cmdc.202100359.
121. S. KB, A. Kumari, D. Shetty, E. Fernandes, C. DV, J. Jays, M. Murahari. (2020). Structure-based pharmacophore modelling approach for the design of azaindole derivatives as DprE1 inhibitors for tuberculosis. Journal of Molecular Graphics and Modelling, 101, 107718. <https://doi.org/10.1016/j.jmgm.2020.107718>.
122. Prohaska, R., Schenkel-Brunner, H. (1975). A simple and efficient method for the preparation of GDP-fucose. Analytical Biochemistry, 69(2), 536-544. https://doi.org/10.1016/0003-2697(75)90158-X.
123. Van Crevel, R., Kleinnijenhuis, J., Oosting, M., Joosten, L. A. B., Netea, M. G. (2011). Innate immune recognition of Mycobacterium tuberculosis. Clinical and Developmental Immunology. https://doi.org/10.1155/2011/405310.
124. Mortaz, E., Adcock, I. M., Tabarsi, P., Masjedi, M. R., Mansouri, D., Velayati, A. A., Casanova, J. L., Barnes, P. J. (2015). Interaction of pattern recognition receptors with Mycobacterium tuberculosis. Journal of Clinical Immunology, 35, 1-10. https://doi.org/10.1007/s10875-014-0103-7.
125. Underhill, D. M., Ozinsky, A., Smith, K. D., Aderem, A. (1999). Toll-like receptor-2 mediates mycobacteria-induced proinflammatory signaling in macrophages. Proceedings of the National Academy of Sciences of the United States of America, 96, 14459-14463. https://doi.org/10.1073/pnas.96.25.14459
126. Means, T. K., Wang, S., Lien, E., Yoshimura, A., Golenbock, D. T., Fenton, M. J. (1999). Human toll-like receptors mediate cellular activation by Mycobacterium tuberculosis. Journal of Immunology, 163, 3920-3927.
127. Girardin, S. E., Boneca, I. G., Viala, J., Chamaillard, M., Labigne, A., Thomas, G., Philpott, D. J., Sansonetti, P. J. (2003). Nod2 is a general sensor of peptidoglycan through muramyl dipeptide (MDP) detection. Journal of Biological Chemistry, 278, 8869-8872. https://doi.org/10.1074/jbc.C200651200.
128. Kundu, I., Paul, G., Banerjee, R. (2018). A machine learning approach towards the prediction of protein-ligand binding affinity based on fundamental molecular properties. RSC Advances, 8, 12127-12137. https://doi.org/10.1039/C8RA00003D.
129. Killick, K. E., Ní Cheallaigh, C., O'Farrelly, C., Hokamp, K., Machugh, D. E., Harris, J. (2013). Receptor-mediated recognition of mycobacterial pathogens. Cellular Microbiology, 15, 1484-1495. https://doi.org/10.1111/cmi.12161.
130. Eric, G., Uzairu, A., Mamza, P. (2016). A Quantitative Structure-activity Relationship (QSAR) Study of the Anti-tuberculosis Activity of Some Quinolones. Journal of Scientific Research and Reports, 10, 1-15. DOI: 10.9734/JSRR/2016/23176.
131. Bose, P., Mishra, M., Gajbhiye, A., Kashaw, S. K. (2019). QSAR, pharmacophore mapping and molecular docking of 2,4-diaminoquinazoline as antitubercular scaffold: A computational hybrid approach. Indian Journal of Pharmaceutical Sciences, 81, 1078-1088. DOI: 10.36468/pharmaceutical-sciences.606.
132. Bhardwaj, S., Dubey, S. (2017). QSAR and Molecular Interaction Study of Piperine Analogues for Antitubercular Activity. Organic & Medicinal Chemistry International Journal, 3, 1-14. DOI: 10.19080/OMCIJ.2017.03.555606.
133. Choudhury, C., Bhardwaj, A. (2020). Hybrid Dynamic Pharmacophore Models as Effective Tools to Identify Novel Chemotypes for Anti-TB Inhibitor Design: A Case Study with Mtb-DapB. Frontiers in Chemistry, 8, 1-13. https://doi.org/10.3389/fchem.2020.596412.
134. Panigrahi, D., Mishra, A., Sahu, S. K. (2020). Pharmacophore modelling, QSAR study, molecular docking and insilico ADME prediction of 1,2,3-triazole and pyrazolopyridones as DprE1 inhibitor antitubercular agents. SN Applied Sciences. https://doi.org/10.1007/s42452-020-2638-y.
135. Joshi, S., Dixit, S., More, U., Kumar, D., Aminabhavi, T., Kulkarni, V. (2014). 3D-QSAR studies of quinoline Schiff bases as enoyl acyl carrier protein reductase inhibitors. Research and Reports in Medicinal Chemistry, 59-75. https://doi.org/10.2147/RRMC.S70339.
136. Hussain, K., Ismael, S., Radhi, W. (2016). Quantitative Structure-activity Relationships (QSAR) and Docking Studies on Pyrimidine Derivatives for Antitubercular Activity against M. tuberculosis H37Rv. British Journal of Pharmaceutical Research, 13, 1-11. DOI: 10.9734/BJPR/2016/27274.
137. Gomes, M. N., Braga, R. C., Grzelak, E. M., Neves, B. J., Muratov, E., Ma, R., Klein, L. L., Cho, S., Oliveira, G. R., Franzblau, S. G., Andrade, C. H. (2017). QSAR-driven design, synthesis, and discovery of potent chalcone derivatives with antitubercular activity. European Journal of Medicinal Chemistry, 137, 126-138. https://doi.org/10.1016/j.ejmech.2017.05.026.
138. Saikia, S., Bordoloi, M. (2019). Molecular Docking: Challenges, Advances and its Use in Drug Discovery Perspective. Current Drug Targets, 20, 501-521. https://doi.org/10.2174/1389450119666181022153016.
139. Ma, J., Sheridan, R. P., Liaw, A., Dahl, G. E., Svetnik, V. (2015). Deep neural nets as a method for quantitative structure-activity relationships. Journal of Chemical Information and Modeling, 55, 263-274. https://doi.org/10.1021/ci500747n.
140. Aleksandrov, A., Myllykallio, H. (2019). Advances and challenges in drug design against tuberculosis: application of in silico approaches. Expert Opinion on Drug Discovery, 14, 35-46. https://doi.org/10.1080/17460441.2019.1550482.
141. Maltarollo, V. G., Kronenberger, T., Wrenger, C., Honorio, K. M. (2017). Current trends in quantitative structure-activity relationship validation and applications on drug discovery. Future Science OA, 3, 3-5. https://doi.org/10.4155/fsoa-2017-0052.
142. Sabitov, L. S., Kashapov, N. F., Strelkov, Y. M., Kuznetsov, I. L. (2017). Method of the steel aluminum wires protection of air transmission lines from fast vibration damage at the output from the support clamp. IOP Conference Series: Materials Science and Engineering, 240, 248-259.
143. Akhter, M. (2016). Challenges in docking: Mini review. JSM Chemistry, 4, 1025.
144. Prieto-Martínez, F., Arciniega, M., Medina-Franco, J. (2018). Acoplamiento Molecular: Avances Recientes y Retos. TIP Revista Especializada En Ciencias Químico-Biológicas. https://doi.org/10.22201/fesz.23958723e.2018.0.143.
